# Disrupting Lipid Raft Microdomains to Block Polyploid Giant Cancer Cell Budding and Enhance Radiotherapy Response

**DOI:** 10.1002/advs.202519698

**Published:** 2025-12-02

**Authors:** Zheng Deng, Haoran Sun, Jin Cheng, Ruyi Zhao, Jianzhu Xie, Yanwei Song, Yucui Zhao, Chenwei Lin, Binjie Hu, Yanping Gong, Jun Lin, Sijia He, Yuntao Luo, Minghui Zhao, Yiwei Wang, Ming Jiao, Yuqin Yang, Jikun Li, Shujie Xia, Chuanyuan Li, Qian Huang

**Affiliations:** ^1^ Cancer Center Shanghai General Hospital Shanghai Jiao Tong University School of Medicine Shanghai 201620 China; ^2^ Shanghai Key Laboratory for Pancreatic Diseases Shanghai General Hospital Shanghai Jiao Tong University School of Medicine Shanghai 201620 China; ^3^ Department of Urology Shanghai General Hospital Shanghai Jiao Tong University School of Medicine Shanghai 200080 China; ^4^ Department of Vascular Surgery Shanghai General Hospital of Nanjing Medical University Shanghai 201620 China; ^5^ Department of Pathology Shanghai General Hospital Shanghai Jiao Tong University School of Medicine Shanghai 200080 China; ^6^ Clinical Microbiology Laboratory Shanghai Center for Clinical Laboratory Shanghai 200126 China; ^7^ Department of Laboratory Animal Center Shanghai General Hospital Shanghai Jiao Tong University School of Medicine Shanghai 201620 China; ^8^ Department of General Surgery Shanghai General Hospital Shanghai Jiao Tong University School of Medicine Shanghai 201620 China; ^9^ Institute for Molecular and Cellular Therapy Chinese Institutes for Medical Research and School of Basic Medicine Capital Medical University Beijing 100069 China

**Keywords:** Budding, Polyploid giant cancer cells, Radio‐sensitization, Radiotherapy, Tumor repopulation

## Abstract

Radiotherapy failure often arises from tumor repopulation by treatment‐resistant cancer cells. Following irradiation, cancer cells can undergo endoreplication to form polyploid giant cancer cells (PGCCs)—radiation‐persistent cells capable of generating progeny through a virus‐like asymmetric budding process. While such membrane budding is evolutionarily conserved across archaea, viruses, and eukaryotic cells, its molecular mechanism in cancer remains poorly defined. Here, a radiation‐induced SNCG–FLOT2–CHMP4B signaling axis is identified as a key regulator of PGCC budding. Mechanistically, ASAH1 and SMPD2 maintain sphingolipid metabolic balance, while FLOT2 drives germination at lipid raft–enriched membrane microdomains, followed by CHMP4B‐dependent abscission to release daughter cells. Disrupting these lipid raft structures—via statins or anti‐PCSK9 antibodies—impairs budding, suppresses PGCC‐derived tumor repopulation, and enhances radiosensitivity in vitro and in vivo. This findings uncover a conserved membrane remodeling program underlying PGCC budding and establish lipid raft disruption as a promising therapeutic approach to prevent tumor recurrence after radiotherapy. Clinically available lipid‐lowering agents may thus serve as innovative radiosensitizers to improve radiotherapy outcomes.

## Graphical abstract

Radiation induces polyploid giant cancer cells (PGCCs) that regenerate tumors through virus‐like budding. This process depends on a SNCG–FLOT2–CHMP4B signaling axis functioning in lipid raft microdomains. Disrupting these domains using statins or anti‐PCSK9 antibodies blocks PGCC budding, suppresses tumor repopulation, and enhances radiotherapy efficacy in vitro and in vivo.

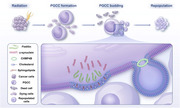

## 1 Introduction

Radiotherapy (RT) remains a cornerstone of comprehensive cancer treatment.^[^ ^1^ ^]^ However, its efficacy is often limited by tumor radioresistance, with tumor repopulation is recognized as a major contributing factor. While many studies have explored this process from the perspectives of cancer stem cells, hypoxia, and tumor‐associated macrophages or fibroblasts, our previous work was the first to report that radiation‐induced apoptotic and necrotic cells could promote the repopulation of neighboring surviving tumor cells through paracrine signaling.^[^ ^2^, ^3^, ^4^ ^]^ We also identified unconventional roles for caspase‐3 and HMGB1 in mediating this effect. Despite these insights, several critical questions remain unanswered: Which types of cancer cells can survive RT? How do these surviving cancer cells initiate tumor repopulation? And could targeting this process enhance the overall effectiveness of RT?

RT eliminates tumor cells primarily by inducing extensive DNA damage, particularly DNA double‐strand breaks. In response, cells activate the DNA damage response to initiate repair processes. If repair is successful, cells may re‐enter the cell cycle; however, incomplete or defective repair can lead to various forms of cell death. During the course of RT, although a substantial number of cancer cells undergo cell death, a distinct population was observed to survive, characterized by progressive cellular enlargement and the presence of either multiple irregular nuclei or a single giant nucleus with a complex karyotype. These cells are referred to as polyploid giant cancer cells (PGCC).^[^ ^5^, ^6^ ^]^ PGCCs primarily arise through endoreplication ^[^ ^5^ ^]^ and were traditionally viewed as genetically unstable cells destined for senescence or mitotic catastrophe. However, emerging evidence suggests a more insidious role for PGCCs. They have been described as a Trojan horse ^[^ ^5^ ^]^ and the evil roots of cancer,^[^ ^7^ ^]^ highlighting their potential to drive tumor repopulation and contribute to therapeutic resistance, thereby posing a significant challenge to effective cancer treatment. Pienta et al.^[^ ^8^ ^]^ proposed that therapeutic resistance driven by polyploidization represents a hallmark of lethal cancer. This concept suggests that the formation of polyploid cells—an evolutionary byproduct of ancestral survival mechanisms—confers distinct advantages under therapeutic stress. In our previous work, we were the first to identify radiation‐induced PGCCs as radiation‐tolerant persister (RTP) cells, characterized by a transient state of radiation tolerance. These RTP cells either ultimately undergo cell death or survive by generating progeny through a budding‐like process. Notably, when tumors repopulated by these progeny cells are subjected to a second round of irradiation, PGCCs can form again, indicating a recurring polyploidization program that may contribute to sustained radioresistance.^[^ ^6^ ^]^

Although PGCCs have been implicated in resistance to oncologic therapies, the mechanisms by which they function remain poorly understood. Given that PGCCs typically harbor tenfold or greater chromosomal ploidy and exist in a state of genetic instability, they are generally incapable of undergoing conventional mitosis.^[^ ^9^ ^]^ However, polyploidy is not an irreversible state. We previously reported that a subset of PGCCs could escape dormancy and give rise to proliferative, parent‐like progeny through an extremely asymmetric, virus‐like budding mechanism, as demonstrated by single‐cell sequencing and functional assays.^[^ ^6^, ^10^ ^]^ These findings suggest that while polyploidy acts as a protective adaptation to stress, budding represents a distinct, non‐mitotic mechanism by which PGCCs generate progeny and contribute to tumor repopulation.^[^ ^9^ ^]^ Therefore, targeting the budding process of PGCCs may represent a promising therapeutic strategy to overcome treatment resistance and suppress tumor repopulation following RT.

Budding is a widely conserved mode of proliferation across biological kingdoms, employed by yeast, enveloped viruses, archaea, and others. In enveloped viruses, budding constitutes the final and most critical step in their replication cycle.^[^ ^11^ ^]^ Liu et al.^[^ ^12^ ^]^ reported that Sulfolobus, a hyperthermophilic and acidophilic archaeon infected by STSV2, undergoes substantial cell enlargement with extensive chromosomal content and abandons binary fission in favor of asymmetric budding to produce original‐sized progeny. Even in eukaryotic cells, processes such as vesicle budding from the plasma membrane and the formation of intraluminal vesicles (ILVs) within multivesicular endosomes (MVEs) rely on budding mechanisms. Although mitosis has largely supplanted budding in higher organisms, this mode of proliferation is not a novel evolutionary phenomenon but rather a primitive strategy that was largely superseded in eukaryotic evolution. Cancer has been proposed as an atavistic condition—a reversion to ancestral cellular programs—where cancer cells do not represent a newly evolved population but instead reactivate ancient survival mechanisms characteristic of unicellular‐like forms.^[^ ^13^, ^14^ ^]^ In this context, budding may represent a primitive, energy‐efficient, and fault‐tolerant alternative to mitosis, enabling the continuation of genetic material under stressful conditions, similar to strategies employed by lower organisms, even if not all progenies are viable.

Based on our hypothesis that budding represents a form of biological atavism, we investigated whether the budding mechanism in tumor cells resembles those observed in enveloped viruses, archaea, and ILVs. In enveloped viruses, budding typically involves hijacking the host's endosomal sorting complexes required for transport (ESCRT) machinery to facilitate maturation and budding at the plasma membrane.^[^ ^15^ ^]^ Similarly, the formation of ILVs and vesicle budding in eukaryotic cells also relies on the highly conserved ESCRT complex.^[^ ^15^ ^]^ In archaea, particularly in *Sulfolobus*, a eukaryotic‐like ESCRT system orchestrates both binary fission and budding events.^[^ ^12^ ^]^ Beyond ESCRT‐dependent pathways, Trajkovic et al.^[^ ^16^ ^]^ identified an ESCRT‐independent pathway for ILV formation within MVEs, which depends on lipid raft microdomains enriched in sphingolipids and sterols. Ceramide plays a key role in this process by promoting the coalescence of small lipid rafts into larger, ordered domains that drive domain‐induced budding.^[^ ^17^ ^]^ Notably, lipid rafts have also been implicated in viral budding.^[^ ^18^ ^]^ Collectively, these findings indicate that lipid rafts and ESCRT complexes emerge as common mechanistic elements in budding processes across biological systems.

This study aimed to elucidate the mechanism of PGCCs budding after RT and to identify therapeutic strategies that target this process to enhance radiosensitivity. Using multiple experimental models—including cancer cell lines, cell line‐derived xenografts, and patient‐derived xenografts (PDX) models—we found that PGCC budding depends on lipid raft–enriched regions of the plasma membrane. Mechanistically, we identified a critical SNCG–lipid rafts (FLOT2, Flotillin‐2)–ESCRT (CHMP4B) axis that drives PGCC budding. Notably, pharmacological disruption of lipid rafts with statins or anti‐PCSK9 antibody effectively suppressed PGCC budding and enhanced radiosensitivity both in vitro and in vivo.

## 2 Results

### 2.1 PGCC Formation and Budding are the Dominant Modes of Tumor Repopulation Following Radiation

We observed the presence of PGCCs and their budding progeny in cultured tumor cells, xenografts, and PDX models, both in vitro and in vivo. Based on these findings, we defined PGCCs as RTP cells. These PGCCs could repopulate tumors through a viral‐budding–like mechanism.^[^ ^6^ ^]^ Whether PGCCs represent the initiating cells for tumor repopulation following RT remains to be fully established, but our findings suggest that further elucidation of the role and significance of PGCC budding in tumor repopulation is warranted.

**Figure 1:**
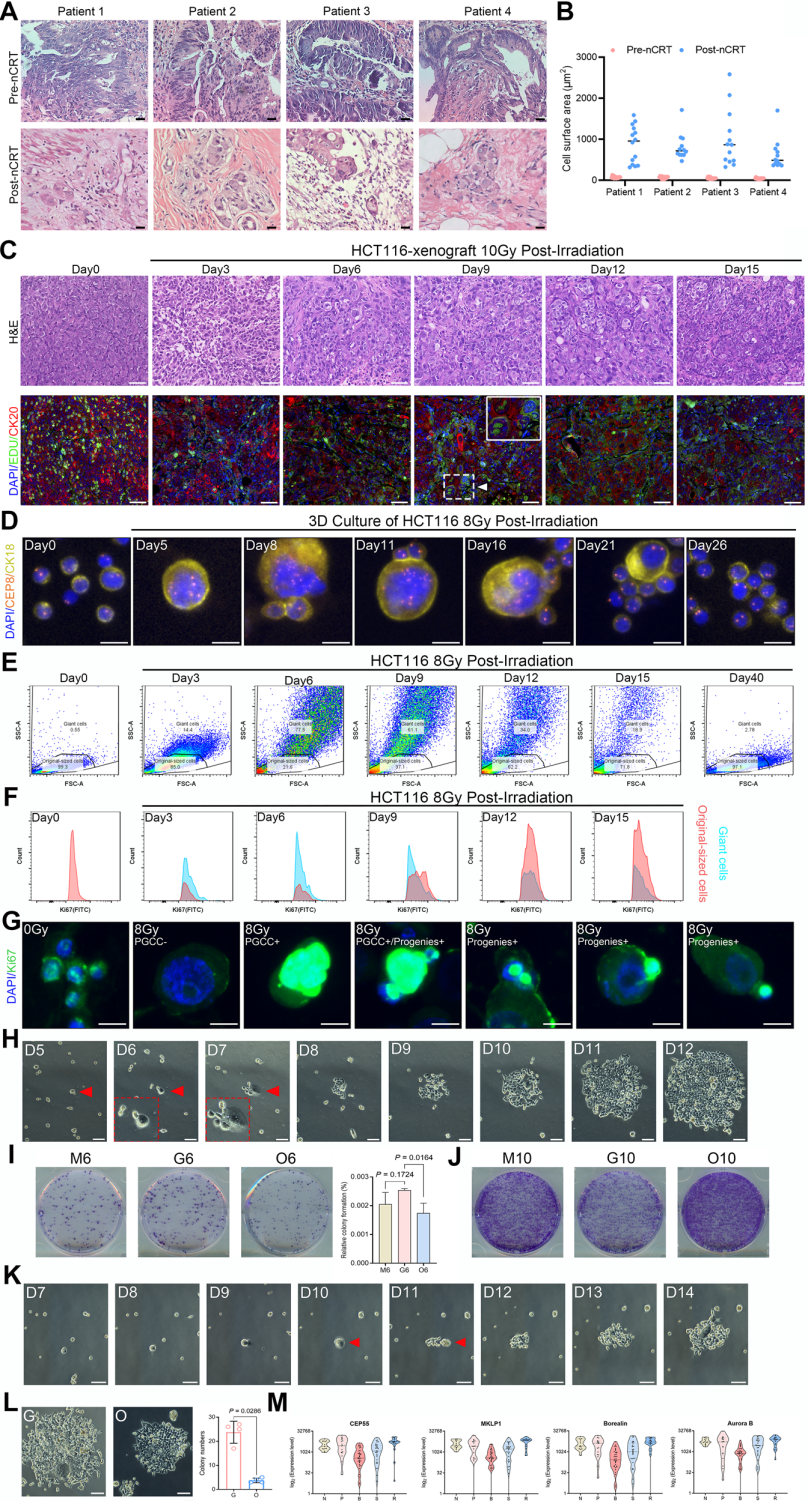
Observation of radiation‐induced PGCC formation and their repopulation. A) H&E staining of four paired pre‐ and post‐nCRT tumor specimens from four patients with rectal cancer (Scale bar, 20 µm). B) Quantification analysis of tumor cell surface area for above four paired pre‐ and post‐nCRT tumor specimens. C) H&E (Upper) and immunofluorescence (Lower) staining for EdU and CK20 showing PGCC formation and proliferative activity in a 10‐Gy‐treated HCT116 xenograft. Scale bar, 50 µm. D) iFISH analysis of suspension‐cultured HCT116 cells showing temporal changes in size and ploidy after 8‐Gy irradiation. Ploidy was assessed using a CEP8 probe which is specific to human chromosome 8 (magenta); DNA and cytokeratin were stained with DAPI (blue) and CK18 (gold), respectively. Scale bars: 20 µm. E) Flow cytometry analysis of cell size which reflected the dynamic changes of PGCC and thereafter new small size cells formation in HCT116 cells post‐irradiation. F) Flow cytometry quantification of Ki67 positive cells in both PGCCs and original‐sized HCT116 cells. G) Represented pictures of smears derived residual samples for Ki67 flow cytometry analysis. Scale bars: 20 µm. H) Time‐lapse tracking the formation of radiation‐induced PGCC formation, PGCC budding (red triangle), and colony formation in HCT116 cells after 8‐Gy irradiation. Scale bars, 100 µm. I) Colony‐forming capacity of HCT116 cells sorted as giant (G6), original‐sized (O6), or unsorted mixture (M6) after 8‐Gy X‐ray irradiation. Cells were analyzed on day 6 post‐irradiation (50000 cells per well, n = 3). Data are presented as mean ± SD. Statistical significance was assessed using the Student's *t*‐test. J) Tracking PGCC formation, PGCC budding (red triangle), and colony formation from original‐sized HCT116 cells sorted on day 6 post‐irradiation (O6). Giant cells began to appear on day 7 and became prominent on day 10 post‐irradiation, indicating progressive enlargement of irradiated cells. Scale bars, 100 µm. K) Original‐sized HCT116 cells sorted on day 6 post‐irradiation were cultured for an additional 4 days. Colony formation of re‐sorted giant (G10), re‐sorted original‐sized (O10), and unsorted mixture (M10) HCT116 cells was then analyzed (50000 cells per well, triplicates for each condition). Giant cells were again obtained and demonstrated the ability to form colonies. L) Comparison of two modes of colony formation in HCT116 cells post‐irradiation: G, colonies involving radiation‐induced giant cancer cells; O, colonies composed exclusively of original‐sized cancer cells. Histogram shows the proportion of these two modes. Data are presented as mean ± SD. Statistical significance was assessed using the Student's *t*‐test. M) Expression of cytokinesis‐related genes (CEP55, MKLP1, Aurora B, and Borealin) in N, P, B, S, and R cells. Expression levels were lowest in budding cells, partially restored in budded cells, and highest in R cells. N, unirradiated cells; P, pre‐budding PGCC cells; B, budding PGCC cells; S, newly budded progenies; R, repopulated cells. iFISH: immunostaining‐fluorescence in situ hybridization; nCRT, neoadjuvant chemoradiotherapy.

To explore whether this process also occurs in human cancer, we examined pathological slides from patients with locally advanced rectal cancer (LARC) who received neoadjuvant chemoradiotherapy (nCRT). Compared to pre‐nCRT biopsy specimens, resection samples obtained 5–6 weeks after nCRT revealed that the residual tumor cells displayed significantly enlarged cell and nuclear sizes (**Figure** **1**A,B). We next analyzed tumor tissues from irradiated xenografts derived from HCT116 human colorectal cancer cells. Hematoxylin and eosin (H&E) staining showed progressive nuclear and cellular enlargement starting at day 3 after irradiation, peaking between days 9 and 12. Immunofluorescence (IF) staining of 5‐ethynyl‐2′‐deoxyuridine (EdU) incorporation revealed that tumor cell proliferation was significantly suppressed by day 3 but gradually resumed by day 6 and became more prominent by day 9. Notably, some enlarged giant nuclei were Edu‐positive (Figure 1C, lower panel, white dotted box), suggesting that a subset of radiation‐induced giant cells remained in a proliferating state (Figure 1C).

To confirm that the giant cells observed in irradiated tumors were indeed PGCCs and their potential proliferative capacity, we performed immunostaining‐fluorescence in situ hybridization (iFISH) and flow cytometry analyses. At first, the dynamic process of formation of giant cell and thereafter new small size cell, we called them original‐sized cells, was observed using iFISH for irradiated HCT116 cells cultured in suspension. To track the changes in size and ploidy in PGCCs and their progenies, the samples at different time point post 8 Gy irradiation were collected (Figure 1D). The giant cells contained 10 or more copies of chromosome 8, consistent with the defining features of PGCCs. In contrast, the progeny derived from budding predominantly harbored two copies of chromosome 8, indicative of diploid cells with size and ploidy nearly identical to those of non‐irradiated parental cells. Then flow cytometry analysis for over 40 days (Figure 1E; Figure S1A, B, Supporting Information) revealed the full timeline of giant cell formation and subsequent tumor cell growth following 8 Gy irradiation. Notably, the proportion of giant cells peaked on day 6, accounting for 77.5% of all live cells. Over time, the number of giant cells sharply declined, while the number of smaller, original‐sized cells increased significantly by day 15, eventually reverting to a cell population resembling the pre‐irradiation state. Flow cytometry analysis confirmed that the Ki67‐positive population was primarily observed in PGCCs within the first six days post‐radiation. As the PGCC population gradually diminished, original sized cells re‐emerged as the dominant Ki67‐positive subset (Figure 1F). The increase in Ki67‐positive rate observed in PGCCs on days 12 and 15 post‐irradiation was attributed to the death of the majority of PGCCs, resulting in a higher proportion of remaining Ki67‐positive PGCCs. Meanwhile, the newly progenies also exhibited elevated Ki67 expression, indicating vigorous proliferative capacity (Figure S1C, Supporting Information). Representative images illustrated both Ki67‐positive and Ki67‐negative staining in PGCCs (Figure 1G). Additionally, we found that daughter cells derived via budding exhibited positive Ki67 staining, indicating that these progeny cells retain proliferation potential.

To investigate the detailed trajectory of tumor cells after irradiation, we first tracked untreated and 8 Gy‐irradiated HCT116 cells at defined time points (Figure S1D, Supporting Information). Unirradiated cells proliferated rapidly, maintained regular morphology and size, and reached near confluence by day 3. In contrast, while irradiated cells also proliferated during the first 2 days, their growth rate subsequently declined, and the cells began to enlarge from day 3 onward. By day 6, a significant increase in giant cells was observed. By day 12, the majority of cells in the tracked fields were PGCCs. We quantified the proportion and area of giant cells in four independent microscopic fields at days 6 and 10 post‐irradiation. Giant cancer cells represented the dominant population, both in terms of cell number and total occupied area (Figure S1E, F, Supporting Information).

To observe the budding behavior of PGCCs, we performed phase‐contrast time‐lapse imaging, which confirmed that PGCCs initially produced progeny through an extremely asymmetrical budding manner. The surviving daughter cells subsequently resumed bipolar division and proliferated rapidly to form colonies (Figure 1H). We next performed a clonogenic assay using three cell populations sorted at day 6 post‐irradiation: giant cells (G6), original‐sized cells (O6), and an unsorted mixed population (M6). The results revealed that G6 exhibited even higher clonogenic potential than that of O6 and M6, although the difference between G6 and M6 was not statistically significant (Figure 1I).

Since PGCCs were observed in colonies derived not only from G6 and M6 but also from O6 (Figure S1G, Supporting Information), this suggested that original‐sized cells might still have the capacity to enlarge and transform into PGCCs. To investigate the fate of these remaining original‐sized cancer cells, we designed the experiment shown in Figure S1H (Supporting Information). Phase‐contrast time‐lapse imaging was performed to trace the destiny of O6 (Figure 1K), and a second clonogenic assay was conducted using cells derived from O6. Specifically, O6 cells maintained in continuous culture were harvested at day 10 post‐irradiation, digested into single cells using trypsin, and separated by mesh filtration. This yielded three populations: sorted giant cells (G10), sorted original‐sized cells (O10), and an unsorted mixed population (M10), which were then seeded into 6‐well plates for clonogenic assays (Figure 1J). Notably, O6 cells gradually increased in size, transformed into PGCCs, and eventually formed colonies through a budding‐like process (Figure 1K). Giant cells were again recovered from the continuous culture of O6‐derived cells (Figure 1J). Notably, the number of colonies produced by O10 and M10 cells was greater than that produced by G10. This was likely due to the presence of small cells from early budded progeny. In other words, O10 and M10 contained small progeny cells derived from asynchronously budding PGCC that had escaped radiation‐induced stress and reverted to mitotic division. Notably, G10 cells—originating from the continuously enlarging O6 population—remained capable of generating new progeny via budding, despite potentially enduring residual radiation‐induced damage. Colonies mediated by PGCCs were still evident in G10, M10, and even O10 populations (Figure S1I, Supporting Information).

To clarify whether PGCCs were the initiators of colony formation post‐irradiation, we utilized daily phase‐contrast time‐lapse tracking images from Figure 1H (covering a total of 110 clones across 4 panoramic scanning fields of unsorted mixed cells) and dynamically followed the clonal formation process from a single irradiated tumor cell to the establishment of a terminal colony. The results indicated that colonies arising solely from original‐sized cancer cells (O) were rare, while giant cancer cells (G) contributed to the majority of colony formation events (Figure 1L). Aurora‐B and Borealin are core components of the chromosome passenger complex (CPC), which is essential for proper chromosome alignment at the metaphase plate and the subsequent separation of sister chromatids. CPC, along with protein regulator of cytokinesis 1 (PRC1) and the RHO GTPase RHOA—which is activated by the guanine nucleotide exchange factor ECT2—collectively regulate cleavage furrow ingression during cytokinesis.^[^ ^19^ ^]^ Notably, CEP55, MKLP1, CPC, and spastin (SPAST) closely interact with the ESCRT machinery through mutual regulatory mechanisms to coordinate cytokinetic abscission. Dysregulation of these genes severely disrupts this critical process.^[^ ^15^ ^]^ To further investigate this mechanism, we analyzed the expression patterns of several cytokinesis‐related genes using our SMART RNA‐sequencing data (GSE221181). We found that their expression was significantly downregulated in budding PGCCs (B), reactivated in newly budded progeny cells (S), and further increased in repopulated cells (R), supporting the notion that PGCCs (B) do not undergo symmetrical mitosis for progeny generation (Figure 1M; Figure S1J, Supporting Information).

In summary, following irradiation, the majority of cancer cells transform into PGCCs as a survival strategy against radiation‐induced stress. These PGCCs bypass conventional mitosis and instead generate progeny through an asymmetrical budding process; the resulting daughter cells progressively regain mitotic competence and contribute to tumor repopulation. PGCC‐mediated budding plays a predominant role in post‐radiation tumor repopulation. Therefore, targeting PGCC budding may represent a critical strategy for suppressing tumor repopulation and enhancing the efficacy of RT.

### 2.2 PGCC Budding Requires Sphingolipid Synthesis

**Figure 2:**
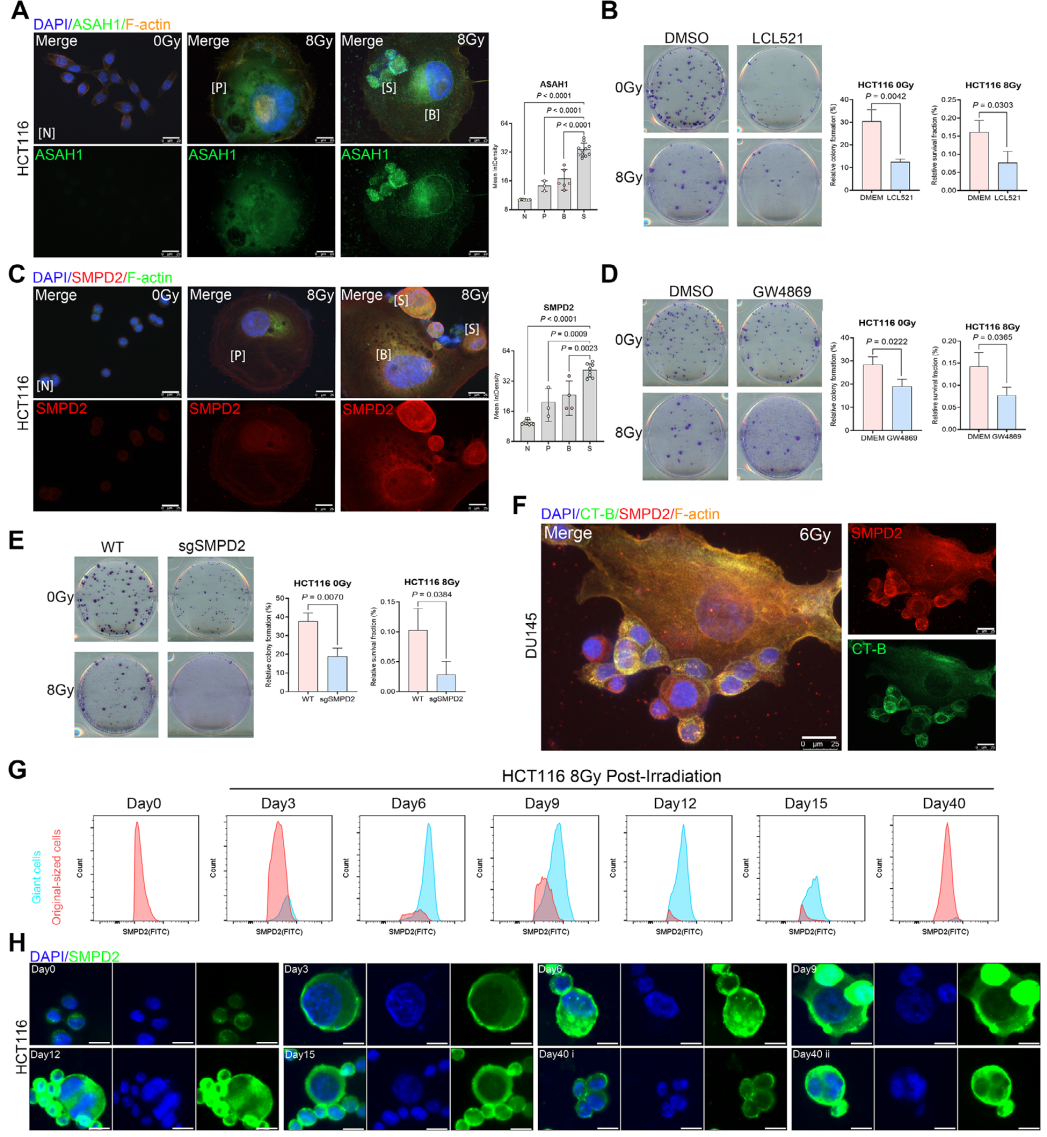
Sphingolipids are involved in PGCC budding. A) Immunofluorescence staining of ASAH1 in unirradiated and radiation‐induced PGCCs in HCT116 cells. The mean integrated density (IntDen) of ASAH1 staining is summarized in the right panel. Scale bar, 25 µm. Data are presented as mean ± SD; statistical significance was assessed using a Student's t‐test. B) Effect of LCL521 (10 µM; ASAH1 inhibitor) on colony formation by unirradiated HCT116 cells (400 cells/well; n = 3 per condition) and 8‐Gy–irradiated HCT116 cells (50000 cells/well; n = 3 per condition). The mean number of colonies (left panel) and mean survival fraction (right panel) are shown. Data are presented as mean ± SD; statistical significance was assessed using a Student's t‐test. C) Representative immunofluorescence image of SMPD2 in HCT116‐derived N, P, B, and S cells. Scale bar: 25 µm. The mean integrated density (IntDen) of SMPD2 staining is summarized in the right panel. Data are presented as mean ± SD; statistical significance was assessed using a Student's t‐test. D, E) Effect of inhibiting nSMase with GW4869 (D, 10 µM; nSMase inhibitor that blocks ceramide production) or knocking out SMPD2 using sgRNA (E) on colony formation by unirradiated HCT116 cells (400 cells/well in the GW4869 group; 200 cells/well in the sgSMPD2 group; n = 3 per condition) and 8‐Gy–irradiated HCT116 cells (50000 cells/well; n = 3 per condition). Data are presented as mean ± SD; statistical significance was assessed using a Student's t‐test. F) Immunofluorescence staining of SMPD2 and CT‐B in irradiated DU145 cells. Scale bar, 25 µm. G) Flow cytometry quantification of SMPD2 fluorescence intensity in both PGCCs and original‐sized HCT116 cells. H) Immunofluorescence analysis combined with flow cytometry showing representative SMPD2 staining at indicated intervals after 8‐Gy irradiation in HCT116 cells. Scale bars: 20 µm. N, unirradiated cells; P, quiescent PGCCs; B, budding PGCCs; S, newly budded progenies.

Our previous study demonstrated that budding is the primary mechanism by which PGCCs generate progeny with proliferative potential; however, the underlying molecular mechanisms remain elusive. As the formation of new cells necessitates membrane biosynthesis, and sphingolipids and cholesterol are major constituents of cellular membranes,^[^ ^20^ ^]^ we explored the role of lipid metabolism in tumor repopulation. Reanalysis of single‐cell transcriptome sequencing data (GSE221181) revealed the activation of several genes associated with membrane lipid metabolism in the budding‐related gene set. Among these, acid ceramidase (ASAH1) emerged as a particularly notable candidate (Figure S2A, Supporting Information). ASAH1 is a key enzyme in sphingolipid metabolism and functions as a cellular rheostat that influences tumor cell fate between survival and death.^[^ ^21^ ^]^ To validate this, IF staining was performed to assess ASAH1 expression in unirradiated cells (N), quiescent PGCCs (P), budding PGCCs (B), and newly budded progeny (S) in HCT116, DU145, and A549 cell lines. ASAH1 expression was significantly elevated in budding PGCCs, with the highest levels observed in newly budded progeny following 6–8 Gy X‐ray irradiation (**Figure** **2**A; Figure S2B, C, Supporting Information). The small‐molecule ASAH1 inhibitor LCL521 suppressed tumor cell growth in a dose‐dependent manner, as assessed by the CCK‐8 assay in vitro (Figure S2D, Supporting Information). Although PGCC budding was still observable at a low concentration of LCL521 (1 µM), no budding was detected at a higher concentration (5 µM) (Figure S2E, Supporting Information). Specifically, while single giant cells exhibiting sprouting dendritic and filamentous extensions remained adherent to the dish, they could not extrude daughter cells or form colonies. Consistently, clonogenic assays confirmed that LCL521 significantly inhibited the clonogenic potential of both non‐irradiated and 8 Gy X‐ray–irradiated tumor cells (Figure 2B; Figure S2F, Supporting Information). These findings suggest that ASAH1 and sphingolipid metabolism are involved in PGCC budding and subsequent tumor repopulation. Furthermore, CRISPR‐mediated knockout (KO) of the ASAH1 gene severely impaired cell survival, indicating an essential role for ASAH1 in tumor cell viability (Figure S2G, H, Supporting Information).

As shown in Figure S2I (Supporting Information), we observed an intriguing phenomenon: budding vesicles with or without nuclei appeared on a single PGCC. Both types of vesicles exhibited similar morphology and elevated ASAH1 expression. This observation led us to hypothesize whether PGCC budding and vesicle formation might share similar underlying mechanisms. A previous study by Trajkovic et al.^[^ ^16^ ^]^ reported that ILV formation may occur via a lipid raft–dependent pathway, in which sphingomyelinase (SMase) promotes ILV budding, while GW4869, a neutral sphingomyelinase (nSMase) inhibitor, produces the opposite effect. To investigate the role of nSMase in PGCC budding, we performed IF staining and found that sphingomyelin phosphodiesterase 2 (SMPD2) displayed a similar expression pattern to that of ASAH1: its levels were significantly elevated in budding PGCCs, particularly in the budded progeny following irradiation (Figure 2C,F; Figure S2J, K, Supporting Information). GW4869 suppressed clonogenic potential in both non‐irradiated and 8 Gy X‐ray–irradiated tumor cells (Figure 2D; Figure S2M, Supporting Information). Furthermore, CRISPR‐mediated KO of SMPD2 impaired colony‐forming capacity (Figure 2E; Figure S2L, Supporting Information), further supporting a critical role of SMPD2 in PGCC‐mediated repopulation.

To further investigate the relationship between SMPD2 expression and the generation of PGCCs and their progeny, we performed time‐resolved flow cytometry and IF staining to monitor changes in SMPD2 expression and cell size throughout a single cycle of PGCC budding following irradiation. Flow cytometry revealed a significant increase in SMPD2 expression in PGCCs, with a more modest elevation observed in original‐sized tumor cells over time (Figure 2G), flow cytometry quantification revealed a transient suppression of SMPD2 positive rate in original‐sized cells during days 12–15 post‐irradiation, with recovery observed by day 40 (Figure S2N, Supporting Information). IF staining was subsequently performed on samples from the same time course, and representative images are shown in Figure 2H. Specifically, by day 6 post‐irradiation, SMPD2 expression was significantly upregulated in PGCCs, and this elevated expression persisted through day 15. By day 40, when the cell population was predominantly composed of original‐sized tumor cells, SMPD2 expression had returned to levels comparable to those in unirradiated controls.

Taken together, these findings suggest that membrane lipid–related proteins, particularly ASAH1 and SMPD2, are required for PGCC budding and tumor cell repopulation. Targeting ASAH1 or SMPD2 may represent a promising strategy to prevent tumor repopulation and enhance the efficacy of RT.

### 2.3 FLOT2 in the Lipid Raft Microdomain is Involved in PGCC Budding

**Figure 3:**
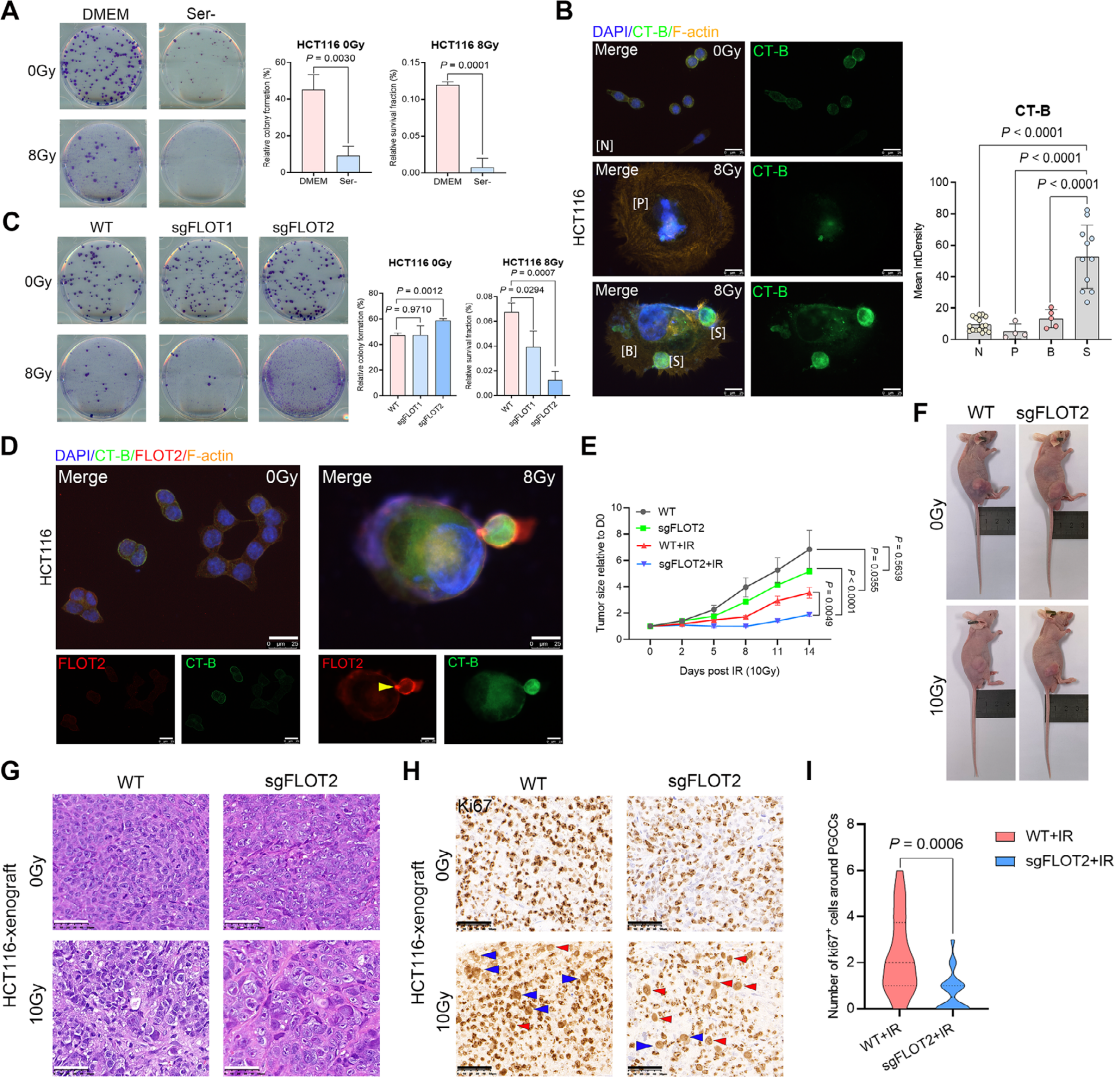
FLOT2 contributes to PGCC budding. A) Effect of serine deprivation on colony formation in unirradiated HCT116 cells (200 cells per well, in triplicate) and 8 Gy‐irradiated HCT116 cells (50000 cells per well, in triplicate). Data are presented as mean ± SD and analyzed using Student's t‐test. B) Representative images (left) and quantification (right) of immunofluorescence staining for CT‐B in unirradiated PGCCs and in radiation‐induced quiescent or budding PGCCs in HCT116 cells. Scale bar, 25 µm. Mean ± SD; Student's t‐test. C) Colony‐forming capacity of wild‐type HCT116 cells and their FLOT1‐ or FLOT2‐knockout derivatives with or without radiation treatment (200 cells per well for 0 Gy and 50000 cells per well for 8 Gy, in triplicate). Data are shown as mean ± SD and analyzed using Student's t‐test. D) Expression of CT‐B and FLOT2 in HCT116 cells in different states. An accumulation of FLOT2 was observed at the junction between PGCCs and their budding progeny cells (yellow arrow). Scale bar, 25 µm. E) Tumorigenic potential assessed in vivo for HCT116 cells and their FLOT2‐knockout derivatives with or without radiation treatment. Day 0 (baseline) was defined as the day radiation was administered. Mean ± SEM, n = 5 per group, two‐way ANOVA. F) Photograph of nude mice bearing xenografts of HCT116 cells or their FLOT2‐knockdown counterparts. G, H) H&E (G) and Ki67 staining (H) showing increased PGCC formation in 10 Gy‐treated HCT116 xenografts. The blue triangle indicates PGCCs with Ki67‐positive cells surrounding them, while the red triangle indicates quiescent PGCCs. Scale bar, 50 µm. I) Violin plots show the number of Ki67‐positive cells surrounding PGCCs in wild‐type and sgFLOT2 plus radiotherapy groups.

Serine serves as a crucial precursor for multiple biosynthetic pathways in tumors. Previous studies^[^ ^17^ ^]^ have demonstrated that serine deprivation impairs the de novo sphingolipid synthesis pathway by affecting serine palmitoyltransferase (SPT) activity, while having minimal impact on nucleotide, redox, and glycerophospholipid metabolism in HCT116 cells. In this study, serine deprivation was employed to disrupt sphingolipid synthesis. As shown in **Figure** **3**A, serine deprivation significantly inhibited colony formation in both unirradiated and irradiated cells. Moreover, PGCCs cultured in serine‐deprived medium displayed aberrant morphology and defective budding (Figure S3A, Supporting Information).

Based on the observations in Figure S2I (Supporting Information), we hypothesized that PGCC budding might share mechanistic similarities with classical budding events observed in biological systems. Lipid rafts—sphingolipid‐ and sterol‐rich liquid‐ordered (Lo) microdomains in the plasma membrane—have been implicated in the formation of ILVs^[^ ^16^ ^]^ and viral budding processes.^[^ ^18^ ^]^ We further investigated whether PGCC budding utilizes raft‐mediated membrane curvature and budding mechanisms. To label lipid raft microdomains, we used Alexa Fluor 488‐conjugated cholera toxin subunit B (CT‐B), a widely recognized probe that binds GM1 gangliosides enriched in lipid rafts. As shown in Figures 2, 3, and Figure S2J–K (Supporting Information), lipid raft microdomains were significantly enriched in budding progeny cells (S). These findings suggest that membrane domains enriched in cholesterol and sphingolipids are closely associated with the PGCC budding process.

Flotillins (FLOTs) are membrane scaffolding proteins localized in lipid raft microdomains, comprising two members—FLOT1 and FLOT2. These proteins promote lipid raft formation, promoting membrane bending, and gradually increasing membrane curvature, leading to budding events.^[^ ^22^ ^]^ A recent study by Wei et al.^[^ ^23^ ^]^ demonstrated the role of lipid rafts and FLOTs protein in ILV budding. They found that active RAB31 first binds to the SPFH domain of FLOTs and subsequently drives ILV formation through inward budding via the FLOT domain—independent of the ESCRT complex. Given the novelty of this mechanism, we investigated the potential involvement of FLOTs in PGCC budding. Western blot analysis revealed that the expression levels of ASAH1, SMPD2, and FLOT2 were upregulated in irradiated tumor cells and gradually declined to baseline in repopulated tumor cells (R) by 40 days post‐irradiation (Figure S3C, Supporting Information). CRISPR‐Cas9‐mediated knockdown of FLOT1 and FLOT2 had no significant influence on clonogenic potential in unirradiated cells but markedly reduced colony formation in cells subjected to 8 Gy X‐ray irradiation (Figure 3C; Figure S3D, E, Supporting Information). Notably, FLOT2 depletion had a more pronounced inhibitory effect on clonogenesis than that of FLOT1, and abnormal, ineffective budding events were frequently observed in FLOT2 KO cells (Figure S3B, Supporting Information). FLOT2 was strongly expressed at membrane junctions between PGCCs and nascent progeny cells where membrane separation was incomplete (Figures 3D and 5E). Furthermore, FLOT2 and lipid rafts were both enriched in (S) cells and showed partial co‐localization (Figure 3D; Figure S3F, G, Supporting Information).

Next, we assessed in vivo tumorigenicity and radiosensitivity of HCT116‐FLOT2 KO cells. Although FLOT2 KO cells were capable of forming tumors in nude mice, their growth was significantly slower than that of wild‐type HCT116 cells, regardless of whether tumors received 10 Gy radiation (Figure 3E,F). Fourteen days post‐irradiation, all mice were sacrificed, and tumor tissues were collected for H&E and Ki67 staining. Partial PGCCs exhibited Ki67 positivity, and tumors derived from wild‐type HCT116 cells showed a higher proportion of Ki‐67–positive cells, particularly small, proliferating cells surrounding PGCCs. In contrast, tumors derived from FLOT2 KO cells exhibited fewer sparse Ki67–positive cells in the observed fields (Figure 3G,H). A comparative image of PGCCs with or without surrounding small cells is presented in Figure 3I. These findings indicate that lipid rafts and FLOT2 are essential for PGCC budding and subsequent repopulation.

### 2.4 Budding in Biological Kingdoms Shares a Conserved Mechanism: ESCRT

The ESCRT machinery is an evolutionarily conserved system that mediates membrane scission events, including cytokinetic abscission, vesicle budding, exosome biogenesis and secretion, viral budding, and even archaeal budding.^[^ ^12^, ^15^ ^]^ In lower organisms, EhVps32—a homolog of human CHMB4B—has been reported to induce phase separation of the plasma membrane, thereby facilitating the formation of lipid rafts.^[^ ^24^ ^]^ This process subsequently promotes membrane curvature at ESCRT‐III–enriched sites, ultimately driving membrane budding.

**Figure 4:**
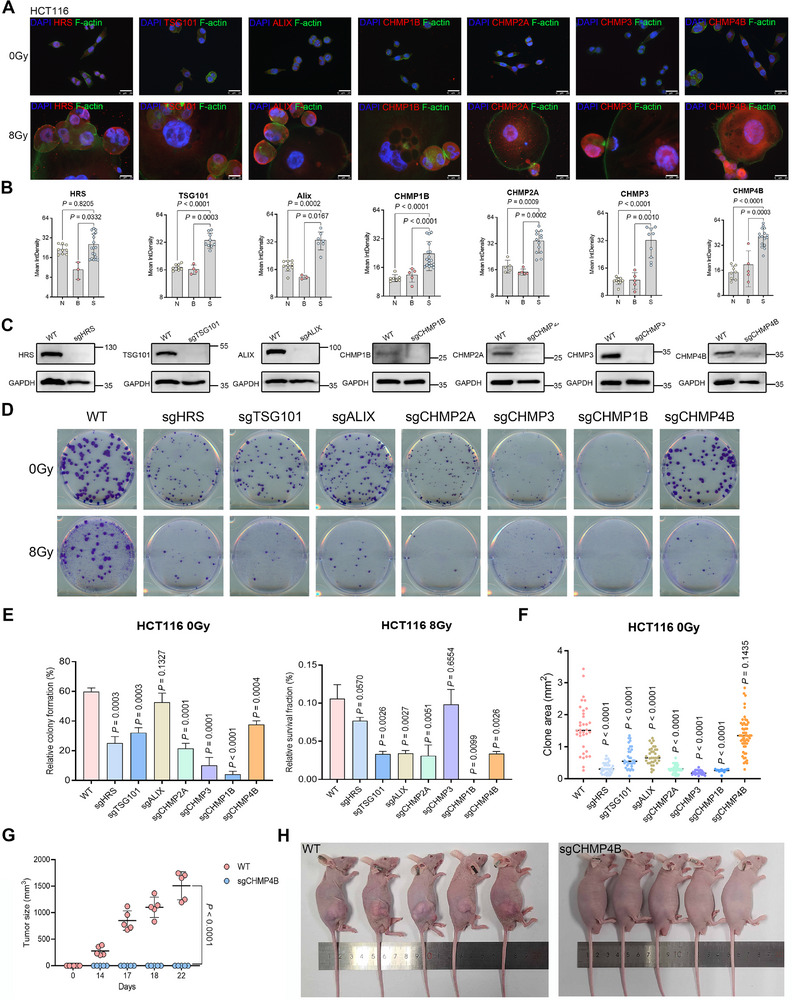
ESCRT components mediate PGCC budding. A) Immunofluorescence staining of ESCRT components, including HRS, TSG101, ALIX, CHMP2A, CHMP1B, CHMP3, and CHMP4B, in unirradiated cells, budding PGCCs, and budded progeny cells derived from HCT116 cells. Scale bar, 25 µm. B) Mean integrated density of ESCRT component immunofluorescence staining. Student's *t‐*test. C) Western blot showing knockout of HRS, TSG101, ALIX, CHMP2A, CHMP1B, CHMP3, and CHMP4B in HCT116 cells. D) Colony‐forming capacity of wild‐type HCT116 cells and corresponding HRS, TSG101, ALIX, CHMP2A, CHMP1B, CHMP3, and CHMP4B knockout cells with or without radiation treatment (200 cells per well for 0 Gy and 50000 cells per well for 8 Gy, n = 3 per condition). E) Mean ± SD of colony numbers and survival fraction. Student's t‐test. F) Dot plot showing surface area of colonies from unirradiated ESCRT component knockout cells. Each point represents one clone. Data are presented as mean ± SD; statistical significance was assessed using a Student's t‐test. In E and F, wild‐type HCT116 cells were used as a control for comparison. G) Tumor growth in nude mice injected with wild‐type HCT116 cells and corresponding CHMP4B‐knockdown cells. Tumor growth kinetics, n = 5 for each group. Mean ± SD, two‐way ANOVA. H) Photograph of nude mice bearing xenografts of HCT116 cells and corresponding CHMP4B‐knockdown cells.

As shown in **Figures** **4**A,B and S4A (Supporting Information), each component of the ESCRT complex exhibited a slightly distinct expression pattern, but all generally followed a budding‐related expression pattern, characterized by relatively higher expression in (S) than in unirradiated cells (N). This suggests that the ESCRT machinery may contribute to the cell budding process. Supporting this notion, ESCRT‐III‐1 has also been reported to be highly expressed in newly generated progeny of STSV2‐infected archaea.^[^ ^12^ ^]^ To further investigate the functional role of ESCRT components in budding, seven ESCRT‐related genes were individually knocked out or knocked down in HCT116 cells using CRISPR‐Cas9 (Figure 4C; Figure S4B, Supporting Information). Clonogenic assays revealed distinct phenotypic effects in non‐irradiated HCT116 cells: CHMP1B and CHMP3 depletion significantly impaired colony formation, while knockdown of HRS, TSG101, and CHMP2A led to moderate reductions (Figure 4D–F). ALIX KO did not significantly reduce the number of colonies but led to a noticeable reduction in colony area. CHMP4B knockdown moderately reduced colony numbers with minimal effect on colony area, likely due to incomplete depletion of CHMP4B protein. However, all seven ESCRT KO or knockdowns—including CHMP4B—exhibited significantly poorer colony formation following irradiation than that of wild‐type HCT116 cells. These findings suggest that disruption of ESCRT components sensitizes tumor cells to radiation, likely by interfering with PGCC budding. In vivo tumorigenicity assays further confirmed this, as CHMP4B‐deficient cells failed to form tumors in nude mice (Figure 4G,H).

This is the first study to report a role for ESCRT in eukaryotic cell budding, a process largely superseded during evolution in favor of mitosis. Under harsh conditions, such as radiation‐ or chemotherapy‐induced stress, PGCCs lose the ability to proliferate via mitosis and instead switch to budding, ensuring the continuity of genetic material. This mode of progeny production represents a more primitive, fault‐tolerant strategy. This budding‐based mode of proliferation in PGCCs may therefore be considered an atavistic reversion to an ancestral eukaryotic state.

### 2.5 SNCG–FLOT2–CHMP4B Axis Regulates PGCC Budding

**Figure 5:**
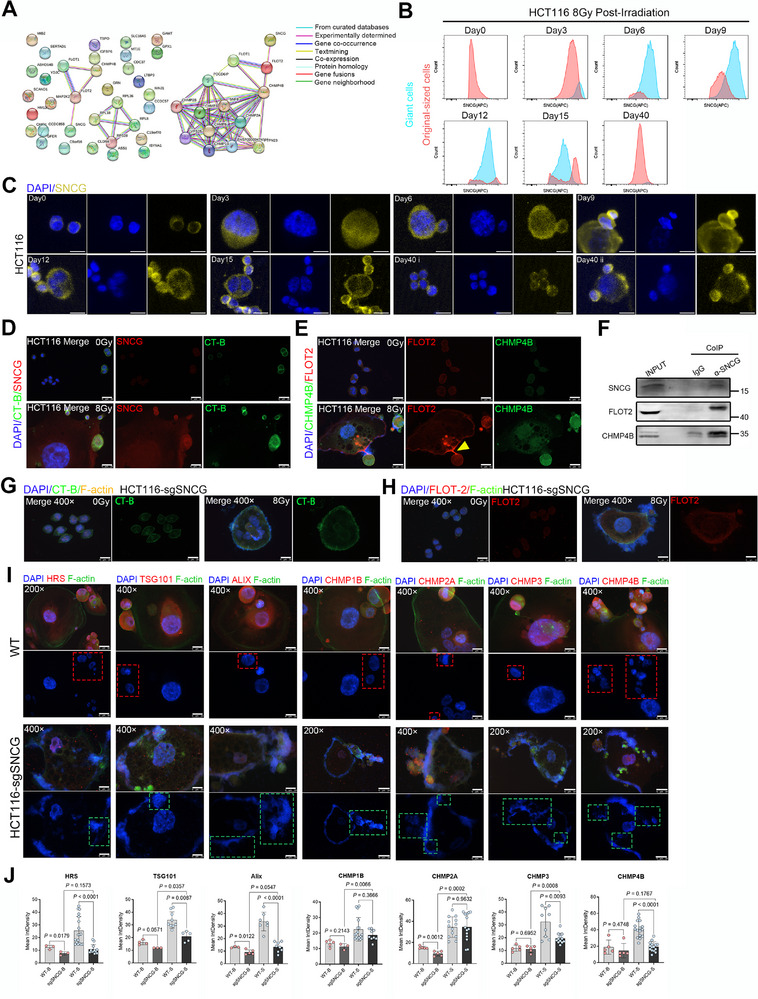
SNCG is an essential factor for PGCC budding. A) Protein–protein interaction network showing SNCG (a budding‐related gene) interacting with FLOT2 and CHMP4B, as predicted by the STRING database. B) Flow cytometric quantification of SNCG fluorescence intensity in PGCCs and original‐sized HCT116 cells. C) Immunofluorescence analysis performed in parallel with flow cytometry showing representative images of SNCG expression at the indicated time points following 8 Gy irradiation in HCT116 cells. Scale bars: 20 µm. D) Immunofluorescence detection of CT‐B and SNCG in untreated and budding HCT116 cells. Scale bars: 50 µm (200×) and 25 µm (400×). E) Immunofluorescence detection of CHMP4B and FLOT2 in untreated and budding HCT116 cells. A distinct accumulation of FLOT2 was observed at the junction between PGCCs and budding progeny cells (yellow arrow). Scale bar: 25 µm. F) Co‐immunoprecipitation of SNCG with FLOT2 and CHMP4B. Proteins were immunoprecipitated using an anti‐SNCG antibody or a non‐specific IgG control, followed by Western blotting with antibodies against FLOT2 and CHMP4B. Results demonstrate that SNCG interacts endogenously with FLOT2 and CHMP4B in HCT116 cells. G, H) Immunofluorescence detection of CT‐B (G) and FLOT2 (H) in untreated and radiation‐induced PGCCs in HCT116‐sgSNCG cells. Scale bar: 25 µm. I) Immunofluorescence staining of ESCRT components (HRS, TSG101, ALIX, CHMP2A, CHMP1B, CHMP3, and CHMP4B) in budding PGCCs and budded progeny cells derived from wild‐type HCT116 and HCT116‐sgSNCG cells. Corresponding DAPI nuclear staining is shown in the lower panels. The red dotted box indicates normal PGCC budding, whereas the green dotted box indicates abnormal budding. Scale bars: 50 µm (200×) and 25 µm (400×). J) Quantification of integrated density (IntDen) for the proteins indicated above in budding PGCCs and budded progeny cells from wild‐type HCT116 and HCT116‐sgSNCG cells after irradiation. Data are presented as mean ± SD. Statistical analysis was performed using Student's t‐test.

γ‐synuclein (SNCG), also known as breast cancer specific gene 1 (BCSG1), was previously identified as a PGCC budding–related gene in our earlier study;^[^ ^6^ ^]^ however, its mechanistic role remained unclear. Based on the budding‐related gene panel established in that study,^[^ ^6^ ^]^ protein–protein interaction analysis via STRING (https://string‐db.org)^[^ ^25^ ^]^ revealed that among the listed genes, only SNCG interacts directly with FLOT2, which associates with the ESCRT protein CHMP4B (**Figure** **5**A).

To further explore this axis, we assessed the relationship between SNCG expression and cell size by flow cytometry. The data showed progressive accumulation of PGCCs accompanied by sustained upregulation of SNCG from day 3 to day 15 post‐irradiation. Although original‐sized cells exhibited a modest increase in SNCG expression, PGCCs displayed significantly elevated levels. Notably, biphasic expression peaks were observed on days 12 and 15. An emerging subpopulation of original‐sized cells with high SNCG expression appeared at day 12, expanded by day 15, and became dominant by day 40, albeit with reduced SNCG expression. These findings reflect the dynamic regulation of SNCG during the transformation of parental cells into PGCCs, their budding progenies, and eventually repopulating cells (Figure 5B), flow cytometry quantification also revealed a significant temporal decline in SMPD2⁺ cell frequency within original‐sized cells clusters at post‐irradiation days 12–15 (Figure S5A, Supporting Information). IF staining confirmed these observations, demonstrating that both radiation‐induced PGCCs and their newly budded progenies exhibited pronounced SNCG overexpression, with a clear correlation between cellular morphology and fluorescence intensity (Figure 5C).

Next, IF staining was performed to validate the expression and subcellular localization of SNCG, FLOT2, and CHMP4B. First, SNCG showed strong co‐localization with lipid rafts and was highly expressed in (S) cells across irradiated HCT116, DU145, and A549 cell lines (Figure 5D; Figure S5B,C,F, Supporting Information). Second, FLOT2 and CHMP4B were highly enriched in (S) cells (Figure 5E; Figure S5D, E, F, Supporting Information). Notably, endogenous protein–protein interactions between SNCG, FLOT2, and CHMP4B were confirmed by co‐immunoprecipitation in HCT116 cells (Figure 5F). These findings are supported by prior studies: Liu et al.^[^ ^26^ ^]^ demonstrated an interaction between SNCG and FLOT, while Hallacli et al.^[^ ^27^ ^]^ revealed that α‐synuclein—which shares a conserved N‐terminal domain with γ‐synuclein—interacts with FLOT and CHMP4B, as identified by quantitative stable‐isotope labeling by amino acids in cell culture mass spectrometry.

Given that SNCG expression was higher in budding PGCCs and newly budded progenies than in quiescent PGCCs or non‐irradiated cells,^[^ ^6^ ^]^ we employed CRISPR‐Cas9 to knock out SNCG and assess its role in PGCC budding (Figure S5G, Supporting Information). SNCG depletion significantly reduced clonogenic survival in both non‐irradiated and irradiated HCT116 cells (Figure S5H, Supporting Information). Notably, SNCG KO cells rarely exhibited the characteristic budding phenotype. Moreover, lipid raft microdomains in PGCCs derived from HCT116‐sgSNCG cells were more unstable (Figure 5G) than those in wild‐type HCT116 cells (Figures 3B and 5D). FLOT2 expression was also significantly downregulated upon SNCG depletion (Figure 5H). We next examined the expression of seven ESCRT proteins in HCT116‐sgSNCG cells. The original budding‐related expression pattern was lost (Figure 5I,J)—specifically, the elevated ESCRT protein levels observed in newly budded progenies of wild‐type cells were no longer present. Notably, in addition to 4′,6‐diamidino‐2‐phenylindole (DAPI) staining in the nuclei, the edges of the PGCC bodies in SNCG‐depleted cells showed DAPI positivity. Some of these signals were round, resembling newly formed nuclei, while others appeared as sharp lines or displayed smeared, ill‐defined boundaries (Figure 5I). Notably, the newly formed progenies lacking nuclei—resembling vesicles generated through abnormal budding—were destined to be non‐viable. This observation suggests that SNCG contributes to lipid raft stability and regulates the expression of FLOT2 and ESCRT proteins during the PGCC budding process. In contrast, the depletion of FLOT2 or ESCRT‐related proteins had minimal impact on SNCG expression (Figure S5I–K, Supporting Information), indirectly indicating that FLOT2 and these ESCRT‐related proteins function downstream of SNCG.

Given the roles of SNCG, FLOT2, CHMP4B, SMPD2, and ASAH1 in PGCC budding, we evaluated their potential as predictors of radiosensitivity using data from The Cancer Genome Atlas (TCGA). However, none of these genes demonstrated strong predictive value for tumor progression in patients with colorectal cancer following RT (Figure S5L, Supporting Information). Similarly, no significant differences in progression‐free survival (PFS) were observed between patients with high versus low expression of these budding‐related genes after RT (Figure S5M, Supporting Information).

Collectively, these findings establish that the SNCG–FLOT2–CHMP4B axis plays a key role in regulating PGCC budding. Disruption of any component of this axis may impair PGCC budding and enhance the therapeutic efficacy of RT.

### 2.6 Disrupting Lipid Rafts on PGCCs with Statins or An Anti‐PCSK9 Antibody Impairs Budding and Improves Radiosensitivity

**Figure 6:**
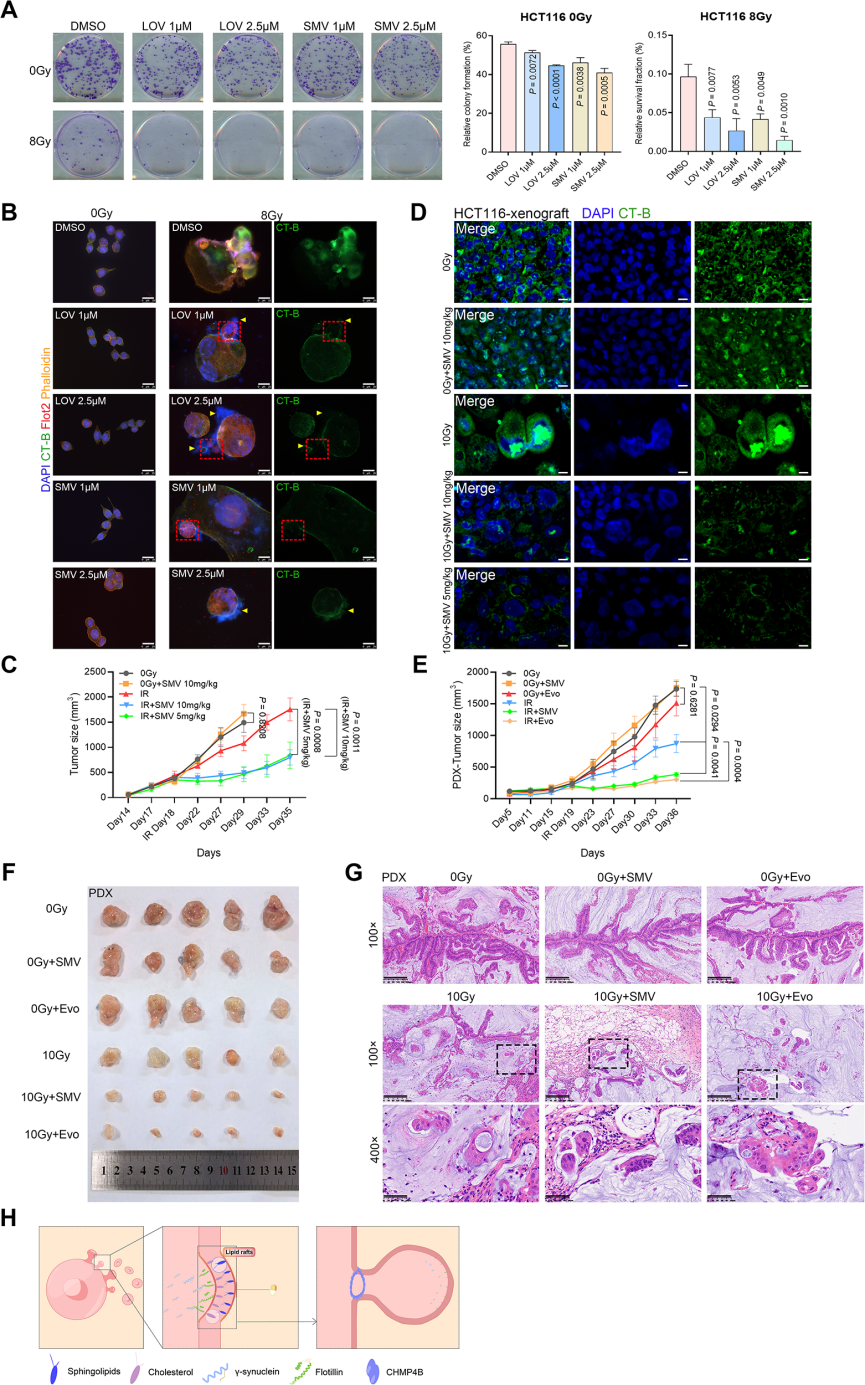
Low‐dose statins and anti‐PCSK9 antibody disrupt lipid rafts of PGCCs and sensitize radiotherapy. A) Effects of lovastatin (LOV) or simvastatin (SMV) on colony formation in unirradiated or 8 Gy‐irradiated HCT116 cells (400 cells per well for 0 Gy and 50000 cells per well for 8 Gy, triplicates for each condition). The mean number of colonies ± SD (left panel) and the mean survival fraction ± SD (right panel) are shown. Student's t‐test. The DMSO group was used as a control for comparison. B) Representative image of immunofluorescence staining suggested that statins interrupt the stabilization of lipid rafts (yellow triangle) and reduce co‐localization between CT‐B and FLOT2 (red dotted box). Scale bar, 25 µm. C) Tumor growth kinetics in HCT116 xenograft model (Mean ± SEM, n = 5 for each group); two‐way ANOVA. D) CT‐B staining showed that SMV remarkably destabilized lipid rafts in the 10 Gy‐treated HCT116 xenograft model. Scale bar, 10 µm. E) Tumor growth kinetics in PDX model (Mean ± SEM, n = 5 for each group); two‐way ANOVA. F) Photograph of PDX tumors. G) H&E staining showed the formation of giant cancer cells in the 10 Gy‐treated PDX model compared to the untreated tumor. Scale bar, 200 µm for 100× and 50 µm for 400×. H) Schematic depicts that process and key component involved in irradiated‐induced PGCC budding.

Blocking the budding represents a promising strategy to enhance the efficacy of RT. While we have partially elucidated the underlying mechanisms of budding, further identification of potential therapeutic targets is required. Drug repurposing in cancer therapy has gained traction, as clinically approved drugs have well‐characterized safety profiles, pharmacodynamics, and toxicities, and their use can reduce the cost of and time required for drug development. Given the critical role of lipid rafts in PGCC budding, these specialized microdomains were selected as therapeutic targets to inhibit tumor repopulation. Previous studies^[^ ^23^, ^28^, ^29^ ^]^ have reported that statins modulate lipid raft dynamics. To evaluate their potential, the half‐maximal inhibitory concentration (IC_50_) of different statins was first determined (Figure S6A, Supporting Information). Clonogenic assays subsequently confirmed that simvastatin (SMV) and lovastatin (LOV) exerted radiosensitizing effects even at low concentrations (**Figure** **6**A). Notably, these statins had a minimal impact on the expression levels of FLOT2, CHMP4B, and SNCG (Figure S6B–C, Supporting Information), suggesting their mechanism of action was independent of these proteins. Based on our previous lineage‐tracing observations, early budding events were first detected on day 6 post‐irradiation. Accordingly, the statin administration schedule was optimized, revealing that a single dose given on day 5 post‐irradiation produced comparable radiosensitizing effects to a two‐dose regimen (on days 1 and 6 post‐irradiation), while minimizing adverse effects on unirradiated cells (Figure S6D, Supporting Information). In clonogenic assays using sorted 8 Gy‐induced giant (G6) and original‐sized (O6) HCT116 cells on day six post‐irradiation, SMV exerted a stronger inhibitory effect on G6 cells than on O6 cells (Figure S6E, Supporting Information). Kaymak et al.^[^ ^30^ ^]^ previously reported that SMV treatment (10 µM) modulated mRNA expression in HCT116 cells and potentially affected cell cycle regulation. However, at a lower concentration (1 µM) of SMV or LOV, no significant changes were observed in the protein expression levels of various cyclins in either irradiated or unirradiated cells (Figure S6F, Supporting Information). Notably, low‐dose statin treatment did not affect unirradiated cells but disrupted the stability of lipid raft microdomains in PGCCs. The co‐localization of lipid rafts and FLOT2 was lost in newly budded daughter cells (Figure 6B), likely due to the high metabolic activity of lipid rafts in PGCCs and their progenies. This may explain why statins enhanced radiosensitivity while exerting minimal effect on unirradiated cells.

The radiosensitizing effect of statins was further validated in HCT116 xenograft tumors. The combination of SMV and irradiation significantly inhibited tumor growth, whereas SMV alone (10 mg kg^−1^) did not delay tumor progression compared to the untreated group (Figure 6C). Moreover, no significant difference was observed between the 5 mg kg^−1^ and 10 mg kg^−1^ SMV combination groups, suggesting that low‐dose SMV was sufficient to achieve effective radiosensitization. In the unirradiated groups, tumor cell morphology was relatively regular regardless of statin treatment, while enlarged and irregular PGCCs were frequently observed in all irradiated groups (Figure S6G, Supporting Information). Ki67 staining revealed that a subset of PGCCs was Ki67–positive, suggesting these cells retained proliferative potential (Figure S6H, Supporting Information). Consistent with in vitro findings (Figure 6B), statins had a minimal impact on lipid rafts in unirradiated tumor cells but significantly disturbed lipid raft integrity in PGCCs in vivo, as evidenced by reduced and irregular CT‐B staining (Figure 6D). In another representative budding field, lipid raft microdomains were active in both budding PGCCs and their progenies in the 10 Gy group (Figure S6I, Supporting Information); however, these domains were severely compromised in the radiation plus SMV group.

PCSK9 is a key regulator protein involved in cholesterol metabolism. Evolocumab (Evo), a clinically approved PCSK9‐neutralizing antibody, is used to lower cholesterol levels in humans^[^ ^31^ ^]^ and also has been shown to be effective in reducing cholesterol in mouse models.^[^ ^32^ ^]^ Liu et al.^[^ ^33^ ^]^ reported that combining anti‐PD1 antibodies with Evo produces a pronounced tumor‐suppressive effect. To investigate the radiosensitizing potential of Evo and statins, a PDX model of colorectal carcinoma was established (Figure 6E,F). While Evo or statin monotherapy had minimal impact on tumor growth, combining either agent with RT significantly enhanced tumor suppression compared to RT alone. PGCCs were also present in the residual PDX tumors following irradiation, exhibiting morphologic changes similar to those observed in human rectal adenocarcinoma specimens treated with neoadjuvant RT (Figures 1A and 6G).

## 3 Discussion

Tumor repopulation is a major barrier to the success of RT; however, the specific cancer cell populations that survive RT and how they mediate repopulation remain poorly understood. In this study, we continuously tracked progression from the formation of PGCCs to the release of their progeny. We also characterized changes in ploidy and cellular morphology. While a substantial number of tumor cells transitioned into the PGCC state after RT, only a small fraction survived, and an even smaller proportion successfully generated progeny through budding. Rajaraman et al. were the first to describe this unconventional form of cell division, termed neosis.^[^ ^34^ ^]^ Our findings establish that PGCCs, via a viral‐mimicry‐like budding process, represent the predominant mechanism of tumor repopulation following radiation. This budding behavior may reflect a form of biological atavism and serves as a critical target for enhancing radiosensitivity.

The pathologist Jinsong Liu proposed several influential concepts in response to the emergence of giant cancer cells following chemotherapy, including the life code, the dualistic origin of human tumors, and McClintock's heredity theory.^[^ ^35^, ^36^, ^37^ ^]^ The enlarged cell size and polyploidy of these cells may confer advantages such as elevated gene expression and enhanced nutrient storage, enabling them to form physical barriers and better withstand genotoxic stress.^[^ ^9^, ^38^ ^]^ Recently, a population of cells known as drug‐tolerant persister (DTP) cells has been described, characterized by a reversible and transient drug‐tolerant state. The PGCCs that arise following irradiation exhibit features consistent with both stress tolerance and phenotypic plasticity. Therefore, by analogy to the DTP state, we refer to these radiation‐induced giant cancer cells as RTPs.^[^ ^6^ ^]^

White‐Gilbertson described PGCCs as giants and monsters, identifying them as a hazardous cellular subpopulation that drives tumor recurrence.^[^ ^39^ ^]^ The World Health Organization (WHO) Classification of Tumors of the Urinary System introduced a novel aggressive variant of acinar adenocarcinoma of the prostate—pleomorphic giant cell adenocarcinoma (PGCA).^[^ ^40^ ^]^ This subtype usually emerged following androgen deprivation therapy or radiotherapy and exhibited cellular morphology remarkably consistent with the PGCC observed in our study. Importantly, the emerged of PGCCs often indicated a poorer clinical outcome,^[^ ^41^ ^]^ PGCCs were positioned as a critical roadblock to antitumor therapeutic efficacy.

Budding is the critical manner mediated by PGCC for tumor repopulation. Various forms of membrane budding not only exhibit similar morphological features but may also share common underlying mechanisms. Singer and Nicolson originally described the plasma membrane as a sea of lipids,^[^ ^42^ ^]^ with denser, more detergent‐resistant, sterol‐ and sphingolipid‐rich regions—known as lipid rafts—floating within it.^[^ ^43^ ^]^ Sphingolipids and cholesterol facilitate lateral segregation of the cell membrane,^[^ ^44^ ^]^ forming the basis for the development of specific Lo phase microdomains—termed lipid rafts. The discovery of the coexistence of cholesterol‐poor, liquid‐disordered (Ld) and cholesterol‐rich, Lo phases represented a major advance in membrane structural biology.^[^ ^28^ ^]^ Recently, growing attention has been directed toward the phenomenon of liquid–liquid phase separation in cell membranes, particularly in relation to its role in membrane curvature and budding events.^[^ ^14^ ^]^ Lipid rafts also serve as dynamic platforms for various cellular processes, including signal transduction, vesicular transport, and cell migration.^[^ ^45^ ^]^ Notably, lipid rafts in tumor cells are more stable and support stronger protein–protein interactions than those in normal cells.^[^ ^46^ ^]^ We confirmed that lipid rafts were highly enriched in newly budded progenies and colocalized with ASAH1 and SMPD2, suggesting that lipids are reassembled and redistributed during the budding process. Sphingomyelinase hydrolyzes sphingomyelin to produce ceramide, whereas acid ceramidase converts ceramide into sphingosine. This reciprocal conversion maintains the dynamic balance of lipid rafts, which is essential for PGCC budding. Disruption of any gene involved in this pathway destabilizes lipid raft metabolism, thereby impairing the budding process. Thus, rather than being regulated by a single sphingolipid‐metabolism‐related gene, PGCCs appear to rely on a common lipid raft–dependent mechanism for membrane budding.

FLOT1 and FLOT2 are highly conserved and ubiquitous expressed proteins that are not only tightly associated with lipid rafts and co‐regulate membrane budding but also contribute to membrane remodeling. Analogous to exosomal and viral budding processes,^[^ ^22^, ^23^ ^]^ we identified a distinct and essential role for FLOTs in PGCC budding. Notably, genetic depletion of FLOT2 significantly impaired PGCC budding and tumor repopulation while also enhancing the therapeutic efficacy of RT in vitro and in vivo. These findings support our hypothesis that PGCC budding depends on the dynamic stability of membrane lipids and FLOT‐enriched microdomains.

The role of ESCRT complexes in ILV budding is well established.^[^ ^15^ ^]^ ESCRT‐I and ESCRT‐II initiate bud formation and stabilize the bud neck, while ESCRT‐III—a dynamic polymer composed of CHMP2A‐B, CHMP3, CHMP4A‐C, and CHMP6—mediates membrane scission. Using IF and clonogenic assays, we found that CHMP4B, a key component of ESCRT‐III, was most strongly associated with PGCC budding, with minimal effects on mitosis. CHMP4B depletion effectively blocked PGCC budding and enhanced the response to RT. Previous studies^[^ ^47^ ^]^ reported that CHMP3 and CHMP1 depletion led to modest reductions in viral release and may influence cell proliferation, whereas CHMP2A and CHMP4B were essential for enveloped viral budding, aligning closely with our results.

Consistent with our hypothesis, both ESCRT machinery and lipid rafts were involved in PGCC budding, resembling mechanisms observed in archaea, ILVs, and viruses. These results support the notion that PGCC budding represents an evolutionarily conserved, atavistic process. Notably, SNCG functioned as a central scaffold for recruiting downstream effectors, including the lipid raft‐associated protein FLOT2 and the ESCRT‐III component CHMP4B, during the budding process. SNCG depletion disrupted the expression pattern of both ESCRT‐ and lipid raft‐associated proteins. Based on these findings, we propose that the SNCG–lipid raft (FLOT2)–ESCRT (CHMP4B) axis constitutes a key regulatory mechanism for PGCC budding. Disrupting any component of this axis effectively blocks budding and sensitizes cells to RT.

Since the entire budding process—from membrane protrusion to progeny release—relies on lipid raft–enriched membranes, we explored whether disrupting lipid rafts could impair PGCC budding and enhance radiosensitivity. Statins, widely used lipid‐lowering agents, are competitive inhibitors of 3‐hydroxy‐3‐methyl‐glutaryl‐CoA reductase (HMGCR), a key enzyme in cholesterol biosynthesis. Previous studies support this approach: Wei et al.^[^ ^23^ ^]^ demonstrated that statin‐induced disruption of lipid raft microdomains inhibited FLOT‐mediated ILV budding, with effects similar to FLOT2 knockdown. Gupta et al.^[^ ^28^ ^]^ used statins to sensitize pancreatic cancer cells to chemotherapy via lipid raft disruption. Additionally, Ono et al.^[^ ^29^ ^]^ reported that cholesterol depletion impaired lipid rafts and blocked HIV‐1 particle release. Building on these findings, we assessed whether statins could disrupt lipid rafts in PGCCs and sensitize them to RT. Statins have previously been used in cancer treatment due to their ability to induce apoptosis, ferroptosis, and autophagy and modulate the tumor microenvironment (TME).^[^ ^48^ ^]^ In our study, low‐dose statins had no significant effect on the cell cycle in either irradiated or unirradiated cancer cells. However, we observed that lipid rafts were highly enriched at the budding sites of PGCCs and within their progeny, both in vitro and in vivo. These lipid raft–rich regions were particularly vulnerable to disruption by low‐dose statins, which selectively impaired PGCC budding without affecting unirradiated cells. Thus, low‐dose statins radiosensitize tumors by specifically targeting the lipid raft–dependent budding process. Moreover, Evo—an anti‐PCSK9 antibody that modulates cholesterol metabolism—also exhibited radiosensitizing effects. Given the established safety of statins and Evo, these agents may offer clinically applicable strategies to enhance the efficacy of RT without additional toxicity.

This study has some limitations. First, the upstream signals that coordinate the activation of SNCG, FLOT2, and CHMP4B in response to radiation remain unclear. Second, the reliance on in vitro and xenograft models may not fully reflect human tumor biology. Third, the long‐term efficacy and safety of statins and Evo as radiosensitizers require further clinical validation. Finally, the fate and tumorigenic potential of budded progeny cells remain to be determined.

Future research should explore the upstream regulation of PGCC budding, investigate the role of lipid metabolism in tumor resistance, and validate these findings in patient‐derived models to improve clinical translation.

In conclusion, we elucidated the mechanism underlying radiation‐induced PGCC budding. Under stress conditions such as RT, tumor cells mount a protective response characterized by the rapid upregulation of SNCG in PGCCs, which becomes enriched at the cell membrane and promotes lipid raft formation. This, in turn, facilitates FLOT2‐driven membrane protrusion. Subsequently, the recruitment of CHMP4B enables scission at the bud neck, allowing the successful release of progeny cells. Disruption of lipid raft stabilization within PGCCs effectively inhibits this atavistic budding process, thereby enhancing the therapeutic efficacy of RT (Figure 6H).

Our findings clarify the molecular basis of PGCC budding and identify statins and Evo as promising radiosensitizing agents. However, budding represents only the executive process of de‐polyploidization. Many aspects of the de‐polyploidization process remain unclear and warrant further investigation. A deeper understanding of PGCC biology will likely uncover additional therapeutic targets for tumor repopulation and advance cancer therapy.

## 4 Experimental Section

### Cell Culture and Irradiation

Human colorectal cancer cells (HCT116, Catalog No: TCHu 99), non‐small cell lung cancer cells (A549, Catalog No: SCSP‐503), prostate cancer cells (DU145, Catalog No: TCHu222), and HEK293T (Catalog No: GNHu44) cells were obtained from the Cell Bank of the Chinese Academy of Sciences (Shanghai, China) in 2020. The cell lines were tested negative for mycoplasma, bacterial contamination. HCT116, A549, and HEK293T cells were maintained in Dulbecco's Modified Eagle Medium, while DU145 cells were cultured in RPMI 1640 medium. All media were supplemented with 10% fetal bovine serum and 1% penicillin–streptomycin (Life Technologies). Cells were incubated at 37 °C in a humidified atmosphere with 5% CO_2_. Each cell line was authenticated and routinely screened for *Mycoplasma* contamination (Yeasen, MycAway one‐step mycoplasma detection kit). X‐ray irradiation was performed using a Varian Clinac iX linear accelerator (Varian Medical Systems, Inc.) at a dose rate of 300 cGy/min.

### Collection of Giant and Original‐Sized Cells

The collection of PGCCs and original‐sized cells was performed as previously described.^[^ ^6^ ^]^ Briefly, irradiated cells were harvested as single‐cell suspensions on day 6 or day 10 post‐irradiation. To isolate original‐sized cells, the suspension was sequentially filtered through 20 µm and 10 µm nylon meshes (Spectrum Laboratories, Inc.). PGCCs were retained on the 20 µm mesh and collected by gentle rinsing with complete medium.

### Colony Formation Assay

Clonogenic survival assays were performed as previously described.^[^ ^49^ ^]^ Cells were seeded in triplicate in 6‐well plates at densities determined according to the radiation dose and cell type: for 0 Gy, 200 or 400 cells per well for HCT116, A549, and DU145 cells; for 8 Gy, 50000 cells/well (HCT116) and 20000 cells per well (A549); and for 6 Gy, 25000 cells/well (DU145). To assess colony formation in sorted subpopulations (giant, original‐sized, and unsorted mixture) isolated on day 6 or day 10 post‐irradiation (8 Gy), 50000 cells/well were plated.

After 10–12 days, colonies were fixed and stained with 0.5% crystal violet, imaged, and manually counted.

### Flow Cytometry Analysis

Cells were harvested by trypsinization, filtered through a 70 µm mesh to generate single‐cell suspensions, and divided into six groups: unstained control, viability‐stained (FVS^+^), isotype control, SMPD2‐stained (SMPD2^+^), SNCG‐stained (SNCG^+^), and triple‐labeled (FVS^+^, SMPD2^+^, and SNCG^+^). Cells were first incubated with fixable viability stain 780 (FVS780; BD Biosciences, RRID: AB_2 869 673) for 15 minutes at 4 °C in the dark, followed by Fc receptor blocking (BD Biosciences, RRID: AB_2 728 082) for 10 minutes at room temperature. Fixation and permeabilization were performed using Fixation/Permeabilization solution (BD Biosciences, RRID: AB_2 869 008) for 20 minutes at 4 °C. Cells were then incubated with anti‐SMPD2 antibody (Proteintech, RRID: AB_3 671 146) or rabbit IgG isotype control (Proteintech, RRID: AB_3 672 282) for 30 minutes at 4 °C. After two washes with Perm/Wash buffer, cells were labeled with fluorescein isothiocyanate (FITC)–conjugated goat anti‐rabbit IgG (Proteintech, RRID: AB_2 890 897) for 30 minutes at 4 °C. Anti‐SNCG antibody (Biotechne, Catalog # NBP3‐20099AF647) was added to the relevant tubes and incubated under the same conditions. All samples were analyzed on a BD FACSCantoII flow cytometer, with a minimum of 10000 events collected per sample. Data were processed using FlowJo software v10.9.0.

### Phase‐Contrast Time‐Lapse Observation of PGCCs Following Irradiation

Time‐lapse phase‐contrast microscopy was performed using a Leica DMi8 inverted microscope equipped with a phase‐contrast objective and LAS X software. HCT116 cells were seeded in 6‐well plates at a density of 50000 cells per well and irradiated with 8 Gy at the designated time point. Morphological changes were observed daily. During image acquisition, the Tile Scan Acquisition Mode was used to maintain the position and scan large areas. Mosaic images were automatically generated using built‐in stitching algorithms. Representative fields were selected and cropped from these mosaics.

### IF Staining, Lipid Raft Labeling, and iFISH

IF analysis was performed as previously described.^[^ ^10^ ^]^ Briefly, treated cells were cultured on coverslips (Fisher Scientific) placed in 24‐well plates. Cells were then fixed with 4% paraformaldehyde for 15–30 minutes, permeabilized with 0.3% Triton X‐100 for 30 minutes, and blocked with 5% bovine serum‐containing blocking buffer for 1 hour at room temperature. Subsequently, cells were incubated with primary antibodies overnight at 4 °C, followed by incubation with fluorochrome‐labeled secondary antibodies (Cell Signaling Technology) for 1 hour at room temperature. The primary antibodies used included: anti‐ASAH1 (Proteintech, RRID: AB_3 085 035), anti‐SMPD2 (Affinity, RRID: AB_2 838 266), anti‐FLOT2 (Santa Cruz Biotechnology, RRID: AB_627 618), anti‐HRS (Santa Cruz Biotechnology, RRID: AB_10 648 901), anti‐TSG101 (Santa Cruz Biotechnology, RRID: AB_671 392), anti‐Alix (Santa Cruz Biotechnology, RRID: AB_673 819), anti‐CHMP1B (Santa Cruz Biotechnology, catalog number: sc‐514013), anti‐CHMP2A (Affinity, RRID: AB_2 844 952), anti‐CHMP3 (Santa Cruz Biotechnology, RRID: AB_2 217 111), anti‐CHMP4B (Affinity, RRID: AB_2 845 061), and anti‐SNCG (Santa Cruz Biotechnology, RRID: AB_1 120 824). Phalloidin (Yeasen) was used for F‐actin staining as required, and nuclei were counterstained with DAPI (Life Technologies). Imaging was performed using a confocal scanning microscope or fluorescence microscope. Quantification of IF signals was conducted using ImageJ software.

Lipid raft staining was performed following the manufacturer's instructions (Invitrogen, V34403). Briefly, cells were incubated with the green‐fluorescent Alexa Fluor 488–conjugated cholera toxin subunit B (CT‐B), which binds specifically to the pentasaccharide moiety of plasma membrane gangliosides localized within lipid rafts, for 15 minutes at 4 °C. After washing, cells were incubated with an anti–cholera toxin subunit B antibody for 20 minutes at 4 °C. Subsequent steps were performed as described for IF staining.

Co‐staining experiments using the iFISH Human CTC Identification Kit (Cytelligen) were conducted with slight modifications. Following sequential immunostaining as described above, cells fixed on coated slides were hybridized with the Vysis Centromere Probe CEP8 Spectrum Orange (Abbott Laboratories) and sealed for hybridization using the S500 Stat Spin ThermoBrite Slide Hybridization System (Abbott Molecular) for 3 hours. For suspension samples, cells were centrifuged and washed with cleaning buffer, then spread onto formatted slides and incubated overnight at 30 °C. Hybridization was subsequently carried out using the same conditions, followed by additional staining with Alexa Fluor 555–conjugated anti‐CK18 antibody (Cytelligen). All images were acquired using a Zeiss Axio Imager Z2 fluorescence microscope.

### CRISPR‐Mediated Gene KO and Lentiviral Infection

CRISPR–Cas9–mediated gene KO was performed as previously reported.^[^ ^50^ ^]^ Single‐guide RNAs (sgRNAs) targeting *ASAH1* (Gene ID: 427), *SMPD2* (Gene ID: 6610), *FLOT1* (Gene ID: 10 211), *FLOT2* (Gene ID: 2319), *CHMP4B* (Gene ID: 128 866), *CHMP2A* (Gene ID: 27 243), *CHMP3* (Gene ID: 51 652), *PDCD6IP/ALIX* (Gene ID: 10 015), *CHMP1B* (Gene ID: 57 132), *TSG101* (Gene ID: 7251), and *HGS/HRS* (Gene ID: 9146) were designed using the online tool from the Broad Institute (https://portals.broadinstitute.org/gpp/public/analysis‐tools/sgrna‐design).^[^ ^51^ ^]^ The sgSNCG‐targeting *SNCG* in HCT116 cells was kindly provided by Dr. Yucui Zhao.^[^ ^6^ ^]^ All sgRNA sequences were listed in Table S1 (Supporting Information). Annealed primers were cloned into the LentiCRISPR v2 vector (Addgene plasmid #52 961), a gift from Dr. Feng Zhang.

CRISPR lentiviral vectors were co‐transfected with packaging plasmids pSPAX2 and pMD2.G into HEK293T cells using Lipofectamine™ 2000 (Invitrogen) following the manufacturer's protocol. Viral supernatants were collected at 48 and 72 hours post‐transfection, filtered through a 0.44 µm membrane, and stored at −80 °C for further use.

Target cells were infected with lentivirus and selected using puromycin (1 µg mL^−1^) for 2 weeks. Surviving cells were seeded into 96‐well plates at a density of 1 cell per well to isolate single clones. Clones were expanded and screened by Western blotting to confirm gene KO. Clones lacking detectable expression of the target protein were used for subsequent experiments.

### Collection of Patient Samples

Formalin‐fixed, paraffin‐embedded tissue specimens were obtained from individuals with LARC treated at Shanghai General Hospital. The study was approved by the Ethics Committee of Shanghai General Hospital (approval number: 2016KY130), and all procedures complied with relevant ethical guidelines. Eligible participants had tumors clinically staged as II–III (cT3/4 and/or N+), and all received nCRT (Table S2, Supporting Information). Long‐course RT was administered at a total dose of 50–53 Gy in 25 fractions, concurrently with capecitabine‐based chemotherapy. Surgical resection was performed 4 weeks following completion of nCRT. Pre‐nCRT tumor biopsy samples were obtained via colonoscopy, while post‐nCRT resection specimens were collected during surgery.

### Mice Experiments

To establish HCT116 tumor xenografts, BALB/c nude mice (male, 4–6 weeks old) were subcutaneously injected with 5 × 10^6^ cells into the right hind leg. When tumor volumes reached approximately 250 mm^3^, mice were exposed to a single 10 Gy dose of X‐ray irradiation (dose rate: 300 cGy/min). Tumor volumes and body weights were measured twice weekly. Tumor volume was calculated using the formula: Volume = 0.5 × length × width^2^. For statin treatment experiments, SMV was administered orally every 2 days, starting on the day of RT. Mice in the 10 Gy + SMV (5 mg kg^−1^) group received 5 mg kg^−1^ of SMV. In the 0 Gy + SMV (10 mg kg^−1^) and 10 Gy + SMV (10 mg kg^−1^) groups, mice were initially given 10 mg kg^−1^ of SMV for three doses, after which the dose was reduced to 5 mg kg^−1^ for the remainder of the study. Mice were euthanized when tumor volumes exceeded 2000 mm^3^ or if signs of significant cachexia were observed.

The PDX model was kindly provided by Dr. Yuqin Yang. For maintenance and propagation, tumor tissues were fragmented and subcutaneously implanted into the right flanks of male BALB/c nude mice (4–6 weeks old). When tumor volumes reached approximately 250 mm^3^, mice received a single 10 Gy dose of X‐ray irradiation (dose rate: 300 cGy min^−1^). For the statin group, SMV was administered orally at 5 mg kg^−1^ every 2 days. For the Evo group, Evo (Amgen) was administered intraperitoneally at a dose of 200 µg every 2 days for the first three doses, then every 4 days thereafter. All drug treatments began on the same day as RT. All animals were housed and maintained by the Shanghai General Hospital Laboratory Animal Center. All animal experiments were performed in accordance with the guidelines of the Animal Care and Use Committee of Shanghai Jiao Tong University School of Medicine (Approval No. 2019‐A020‐01).

### Histopathological Analysis and Immunofluorescence

Tumor tissues were fixed in 10% neutral‐buffered formalin, embedded in paraffin, and serially cut into 4 µm sections for H&E and immunohistochemistry (IHC) staining. For IHC, tissue sections were stained with H&E or incubated with an anti‐Ki67 antibody (1:200 dilution, Cell Signaling Technology, RRID: AB_2 636 984) following the manufacturer's instructions. For in vivo lipid raft staining, tissue sections were processed according to the manufacturer's protocol (Invitrogen, V34403). For EdU proliferation assays, mice were injected intraperitoneally with 50 mg kg^−1^ EdU (BeyoClick™ EdU‐488 kit, Beyotime, C0071L) 16 hours prior to sample collection. EdU detection was then performed in accordance with the manufacturer's instructions.

### Western Blotting and Immunoprecipitation (IP)

Cells were lysed in RIPA buffer (NCM) supplemented with a protease inhibitor cocktail (NCM) for 20 minutes on ice. Protein concentrations were determined using the bicinchoninic acid (BCA) Protein Assay Kit (Thermo Fisher). Samples were then mixed with loading buffer (Beyotime, Jiangsu, China) and boiled at 100 °C for 10 minutes. Proteins were separated via sodium dodecyl sulfate (SDS)‐polyacrylamide gel electrophoresis and transferred to polyvinylidene difluoride (PVDF) membranes (Bio‐Rad, Hercules, CA, USA). Membranes were incubated overnight at 4 °C with primary antibodies, including anti‐ASAH1 (Proteintech, RRID: AB_3 085 035), anti‐SMPD2 (Affinity, RRID: AB_2 838 266), anti‐FLOT1 (Santa Cruz Biotechnology, RRID: AB_210 656), anti‐FLOT2 (Santa Cruz Biotechnology, RRID: AB_627 618), anti‐HRS (Santa Cruz Biotechnology, RRID: AB_10 648 901), anti‐TSG101 (Santa Cruz Biotechnology, RRID: AB_671 392), anti‐Alix (Santa Cruz Biotechnology, RRID: AB_673 819), anti‐CHMP1B (Santa Cruz Biotechnology, catalog number: sc‐514013), anti‐CHMP2A (Affinity, RRID: AB_2 844 952), anti‐CHMP3 (Santa Cruz Biotechnology, RRID: AB_2 217 111), anti‐CHMP4B (Cell Signaling Technology, catalog number: 42 466), anti‐SNCG (Santa Cruz Biotechnology, RRID: AB_1 120 824), anti‐CDK4 (Cell Signaling Technology, RRID: AB_2 631 166), anti‐Cyclin E1 (Cell Signaling Technology, RRID: AB_2 071 200), anti‐Cyclin B1 (Cell Signaling Technology, RRID: AB_2 072 132), anti‐Cyclin A1 + Cyclin A2 (Abcam, RRID: AB_2 885 076), and anti‐GAPDH (ABclonol, RRID: AB_2 769 863). Following primary incubation, membranes were incubated with appropriate secondary antibodies for 2 hours at room temperature. Signal detection was performed using the enhanced chemiluminescence (ECL) Plus reagent (Roche).

For IP, cells were lysed in IP buffer (50 mM Tris‐HCl, pH 7.4, 150 mM NaCl, 1% NP40, 1 mM EDTA) supplemented with a protease inhibitor cocktail. Lysates were cleared by centrifugation at 14000 × g for 10 minutes at 4 °C, and the resulting supernatants were collected. The supernatants were then incubated with anti‐SNCG antibody (Santa Cruz Biotechnology, RRID: AB_1 120 824) or control IgG (ABclonol, RRID: AB_2 771 930, AB_2 770 414), followed by incubation with Protein A/G magnetic beads (MCE, HY‐K0202). Beads were resuspended in loading buffer and heated at 95 °C for 5 minutes. For subsequent IPs, anti‐CHMP4B (Affinity, RRID: AB_2 845 061) and anti‐FLOT2 (Santa Cruz Biotechnology, RRID: AB_627 618) antibodies were used following the same procedure.

### Predictive Ability of Budding‐Related Genes

To evaluate the predictive value of budding‐related genes, data from the TCGA program were utilized, including cases of colon, rectum, and rectosigmoid junction adenocarcinomas (TCGA‐COAD, TCGA‐READ; n  =  35).

Receiver operating characteristic (ROC) curves were generated using the R package “survivalROC,” and the area under the curve (AUC) was calculated to assess predictive performance. Patients were stratified into high‐ and low‐expression groups based on the median expression level of each gene. Survival analysis was conducted using PFS and corresponding follow‐up data.

### Cell Proliferation and Cytotoxicity Assay

Cell proliferation and cytotoxicity were assessed using a cell counting kit‐8 (CCK‐8; Yeasen, 40 203). For the proliferation assay, cells were seeded in 96‐well plates at a density of 1000 cells per well and incubated for 0, 12, 24, 36, 48, or 72 hours. At each time point, 10 µL of CCK‐8 reagent was added to each well and incubated for 2 hours at 37 °C. The absorbance was measured at 450 nm using a microplate reader.

For the cytotoxicity assay, cells were seeded in 96‐well plates at a density of 3000 cells per well and allowed to adhere overnight. Cells were then treated with statins at various concentrations (0.1–100 µM) for 48 or 72 hours. The subsequent steps were performed as described above.

### Statistical Analysis

All data were analyzed using GraphPad Prism 8 (GraphPad Software) and SPSS 13.0.1 (IBM, Armonk, NY). Data was presented as mean ± SD or mean ± SEM from ≥3 biological replicates. Normality was assessed by Shapiro‐Wilk test. Comparisons between two groups were performed using an unpaired Student's t‐test. Multiple group comparisons involving two variables were performed by two‐way ANOVA with Bonferroni's post hoc test. A p‐value < 0.05 was considered statistically significant.

### Ethical Approval

All animal studies were conducted in accordance with the ethical standards of the Animal Care and Use Committee of Shanghai General Hospital, Shanghai Jiao Tong University School of Medicine, China (No. 2019‐A020‐01).

## Conflict of Interest

The authors declare no conflict of interest.

## Author Contributions

Z.D., H.S, J.C., and R.Z. contributed equally to this work. Q.H. and Z.D. performed conceptualization. Z.D., H.S., R.Z., J.X., Y.S., Y.Z., CL., B.H., Y.G., J.L., S.H., Y.L., M.Z., Y.W., M.J., Y.Y., and J.L. performed methodology. Z.D., R.Z., Y.S., Y.Z., J.X., and B.H. performed Software. Z.D., H.S., J.C., and R.Z. performed Validation. Z.D., H.S., and Y.S. performed visualization. Q.H. and Z.D. performed project administration. Q.H., C.L., S.X. performed supervision. Z.D. writing – original draft. Q.H., C.L., J.C. writing – review & editing.

## Supporting information



Supporting Information

Supplementary Table S1

Supplementary Table S2

## Data Availability

SMART RNA‐Sequencing data were from our previously published paper (PMID: 36658726, GSE221181). The human colon, rectum, and rectosigmoid junction adenocarcinoma (TCGA‐COAD, TCGA‐READ) gene expression and clinical information data were derived from the TCGA Research Network: http://cancergenome.nih.gov/. All other data supporting the findings of this study are available in the main text or the supplementary materials.
