# Supplementary material for: Disrupting Lipid Raft Microdomains to Block Polyploid Giant Cancer Cell Budding and Enhance Radiotherapy Response
Source: Adv Sci (Weinh). 2025 Dec 2;13(9):e19698. doi: 10.1002/advs.202519698 (PMC12904004; doi:10.1002/advs.202519698)
Supplement: Supplementary file 1 — Supporting Information [file ADVS-13-e19698-s002.docx]

**Supporting Information**

Disrupting Lipid Raft Microdomains to Block Polyploid Giant Cancer Cell Budding and Enhance Radiotherapy Response

Zheng Deng^a,b,c,1^, Haoran Sun^a,b,1^, Jin Cheng^a,b,1^, Ruyi Zhao^d,1^, Jianzhu Xie^a,b^, Yanwei Song^a,b^, Yucui Zhao^a,b^, Chenwei Lin^c^, Binjie Hu^a,b^, Yanping Gong^a,b^, Jun Lin^e^, Sijia He^a,b^, Yuntao Luo^f^, Minghui Zhao^a,b^, Yiwei Wang^a,b^, Ming Jiao^g^, Yuqin Yang^g^, Jikun Li^h^, Shujie Xia^c,*^, Chuanyuan Li^i,*^, and Qian Huang^a,b,*^

^a^: Cancer Center, Shanghai General Hospital, Shanghai Jiao Tong University School of Medicine, Shanghai 201620, China.

^b^: Shanghai Key Laboratory for Pancreatic Diseases, Shanghai General Hospital, Shanghai Jiao Tong University School of Medicine, Shanghai 201620, China.

^c^: Department of Urology, Shanghai General Hospital, Shanghai Jiao Tong University School of Medicine, Shanghai, 200080, China.

^d^: Department of Vascular Surgery, Shanghai General Hospital of Nanjing Medical University, Shanghai 201620, China.

^e^: Department of Pathology, Shanghai General Hospital, Shanghai Jiao Tong University School of Medicine, Shanghai 200080, China.

^f^: Clinical Microbiology Laboratory, Shanghai Center for Clinical Laboratory, Shanghai, 200126, China.

^g^: Department of Laboratory Animal Center, Shanghai General Hospital, Shanghai Jiao Tong University School of Medicine, Shanghai 201620, China.

^h^: Department of General Surgery, Shanghai General Hospital, Shanghai Jiao Tong University School of Medicine, Shanghai 201620, China.

^i^: Institute for Molecular and Cellular Therapy, Chinese Institutes for Medical Research and School of Basic Medicine, Capital Medical University, Beijing 100069, China.

^*^**:** Corresponding author.

E-mail: huangqian_sjtu@163.com; chuanli@cimrbj.ac.cn; [xsjurologist@163.com](mailto:xsjurologist@163.com)

^1^: These authors contributed equally to this work.


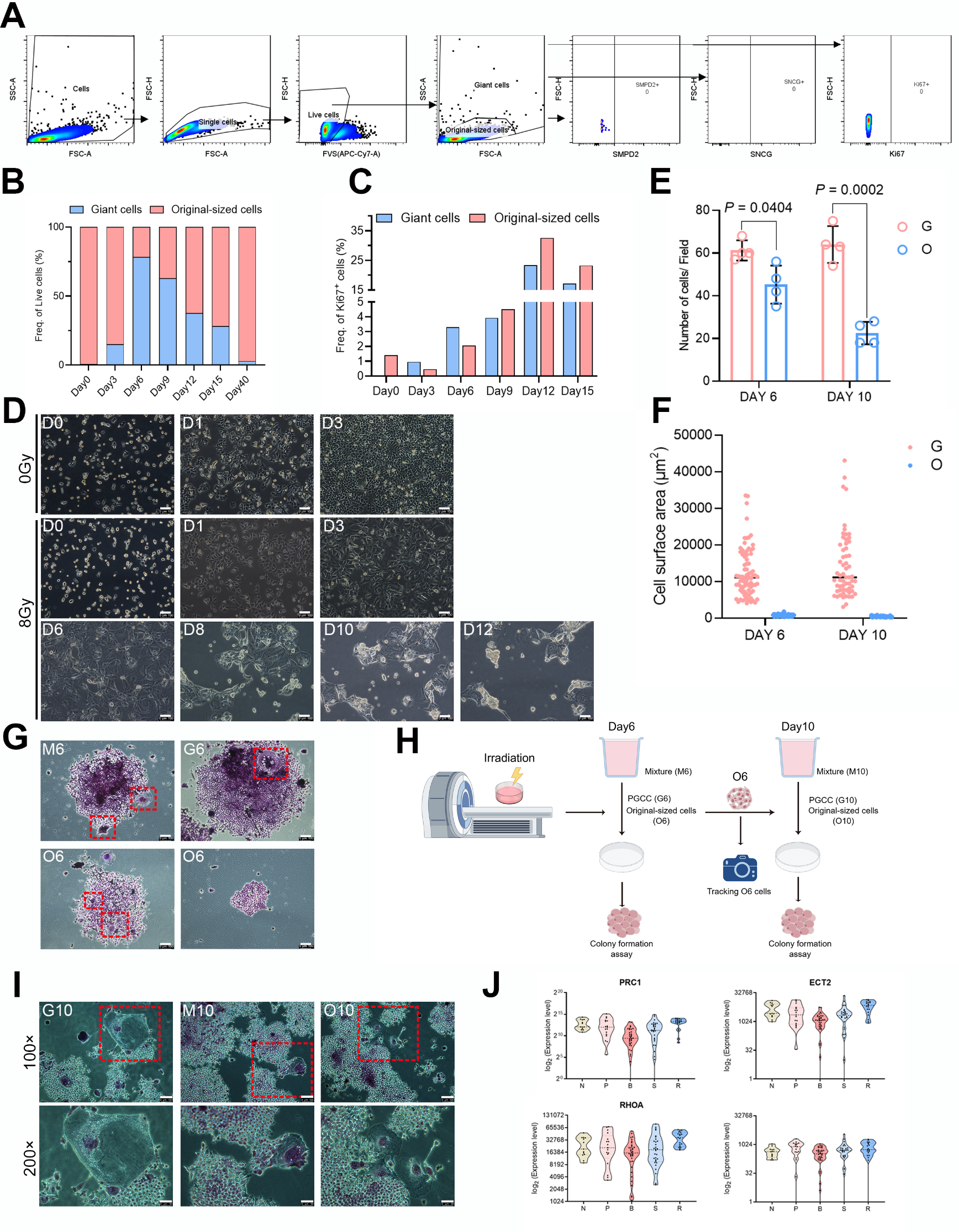


**Figure S1. PGCCs were capable of repopulating *via* budding. A)** Gating strategy for flow cytometry in Figure 1B, Figure 2E, and Figure 5B. Representative example of a population of HCT116, with sequential gates for cells (SSC-A vs. FSC-A), single cells (FSC-H vs. FSC-A), live cells (FSC-H vs. fixable viability stain), giant and original-sized cells (SSC-A vs. FSC-A), SMPD2+ cells (FSC-H vs. FITC), and SNCG+ cells (FSC-H vs. APC). **B)** Quantification of PGCC formation rate of HCT116 cells post-irradiation by flow cytometry. **C)** Flow cytometry quantification of Ki67 positive rate in both PGCCs and original-sized HCT116 cells. **D)** Continuous observation of morphologic changes in untreated or 8 Gy-irradiated HCT116 cells. The pictures were taken from the same field in untreated or 8 Gy-irradiated HCT116 cells. Scale bars, 100 µm. **E-F)** Quantitative analysis of cell number and surface area of giant cells (G) and original-sized cells (O) on day 6 and day 10 post-irradiation, measured in four randomly selected 100× microscopic fields. Data are presented as mean ± SD; Student’s t-test. **G)** Detailed morphology and components in colonies derived from M6, O6, and G6. The giant cell is shown in a red dotted box. Scale bars, 100 µm. **H)** Scheme illustrating the procedure for irradiation and sorting. On day 6 after 8 Gy X-ray irradiation, giant (G6), original-sized (O6), and unsorted mixture (M6) HCT116 cells were isolated for colony formation assays and further culture. On day 10 post-irradiation, the O6 were cultured for 4 additional days, then subjected to a second sorting to obtain giant (G10), original-sized (O10), and unsorted mixture (M10) cells for colony formation assay. The sorting experiment was performed by trypsin digestion into single cells and then filtration by mesh. **I)** Detailed morphology and components of colonies derived from M10, O10, and G10 cells. The giant cell is shown in a red dotted box. Scale bar, 100 µm for 100× and 50 µm for 200×. **J)** Gene expression signatures of cytokinesis-related genes (PRC1, ECT2, RHOA, and SPAST) in N, P, B, S, and R cells. N, unirradiated cells; P, pre-budding PGCC cells; B, budding PGCC cells; S, newly budded progenies; R, repopulated cells.


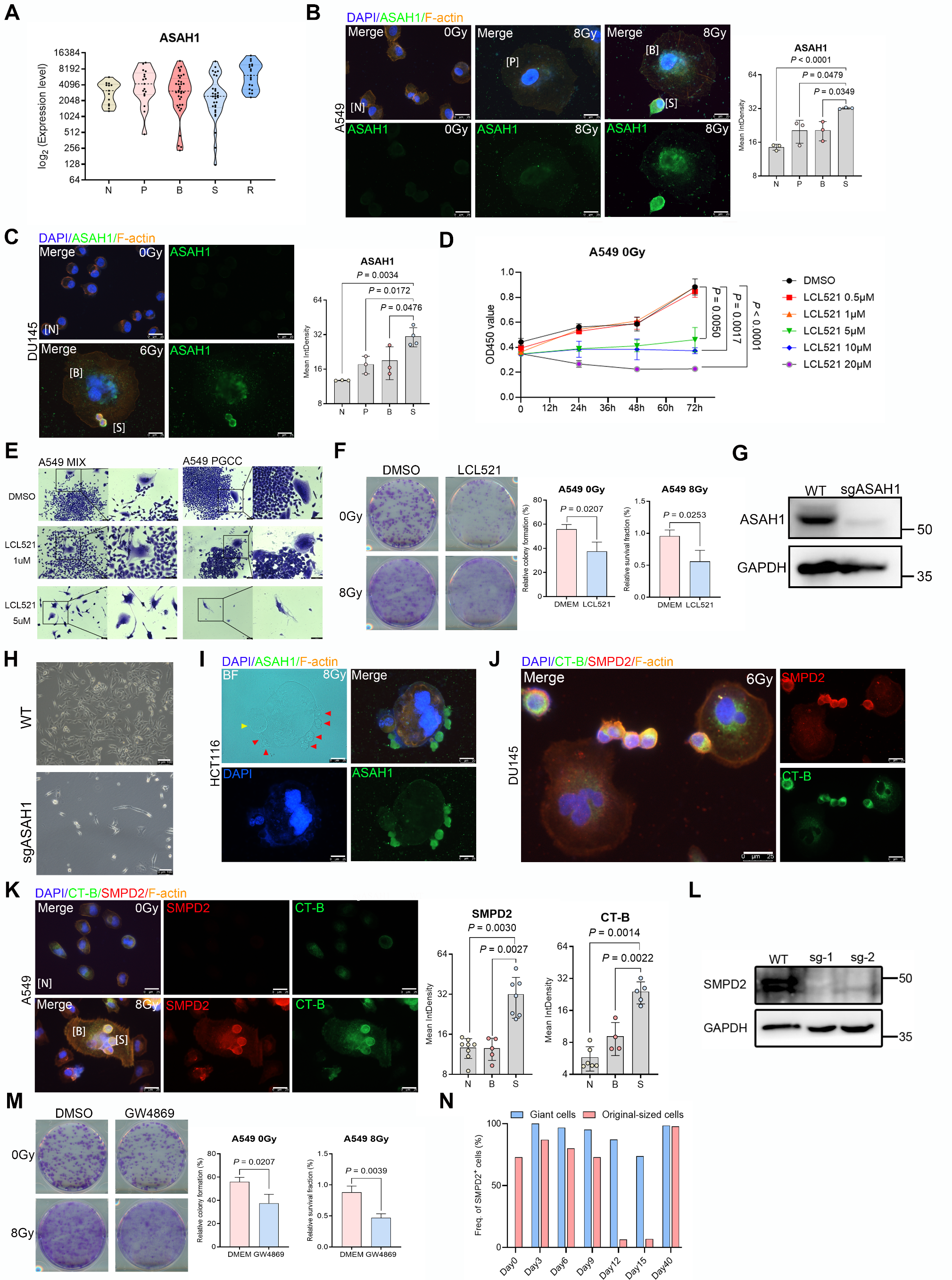


**Figure S2. Sphingolipids are involved in PGCC budding. A)** Expression signatures of ASAH1 in violin plots. **B-C)** Representative images (left) and quantifications (right) of immunofluorescent staining of ASAH1 in A549 (**B**) and DU145 cells (**C**). Scale bar, 25 µm. Data are presented as mean ± SD; Student’s t-test. **D)** Effects of LCL521 concentration on A549 cell proliferation. Data are presented as mean ± SD, n=3; two-way ANOVA. **E)** Representative photographs showing the effect of LCL521 concentration on the budding of PGCCs derived from A549 cells. Scale bar, 100 μm. **F)** Effects of LCL521 (10 μM) on unirradiated (400 cells per well, triplicates for each condition) and 8 Gy-irradiated (20,000 cells per well, triplicates for each condition) A549 cells. Data are presented as mean ± SD; Student’s t-test. **G)** Western blot showing knockout of ASAH1 in A549 cells. **H)** Different morphology in wild A549 and ASAH1-knockout A549 cells. Scale bar, 100 µm. **I)** Immunofluorescent staining showing the expression of ASAH1 not only in budding progenies (with DAPI staining, yellow triangle) but also in budding vesicles (without DAPI staining, red triangle). Scale bar, 25 µm. **J-K)** Expression of CT-B and SMPD2 in DU145 (**J**) and A549 (**K**) cells. Scale bar, 25 µm. Data are presented as mean ± SD; Student’s t-test. **L)** Western blot showing knockout of SMPD2 in HCT116. **M)** Effects of GW4869 (10 μM) on colony formation in unirradiated (400 cells per well, triplicates for each condition) and 8 Gy-irradiated (20,000 cells per well, triplicates for each condition) A549 cells. Data are presented as mean ± SD; statistical significance was assessed using Student’s t-test. **N)** Flow cytometry quantification of SMPD2 positive rate in both PGCCs and original-sized HCT116 cells.


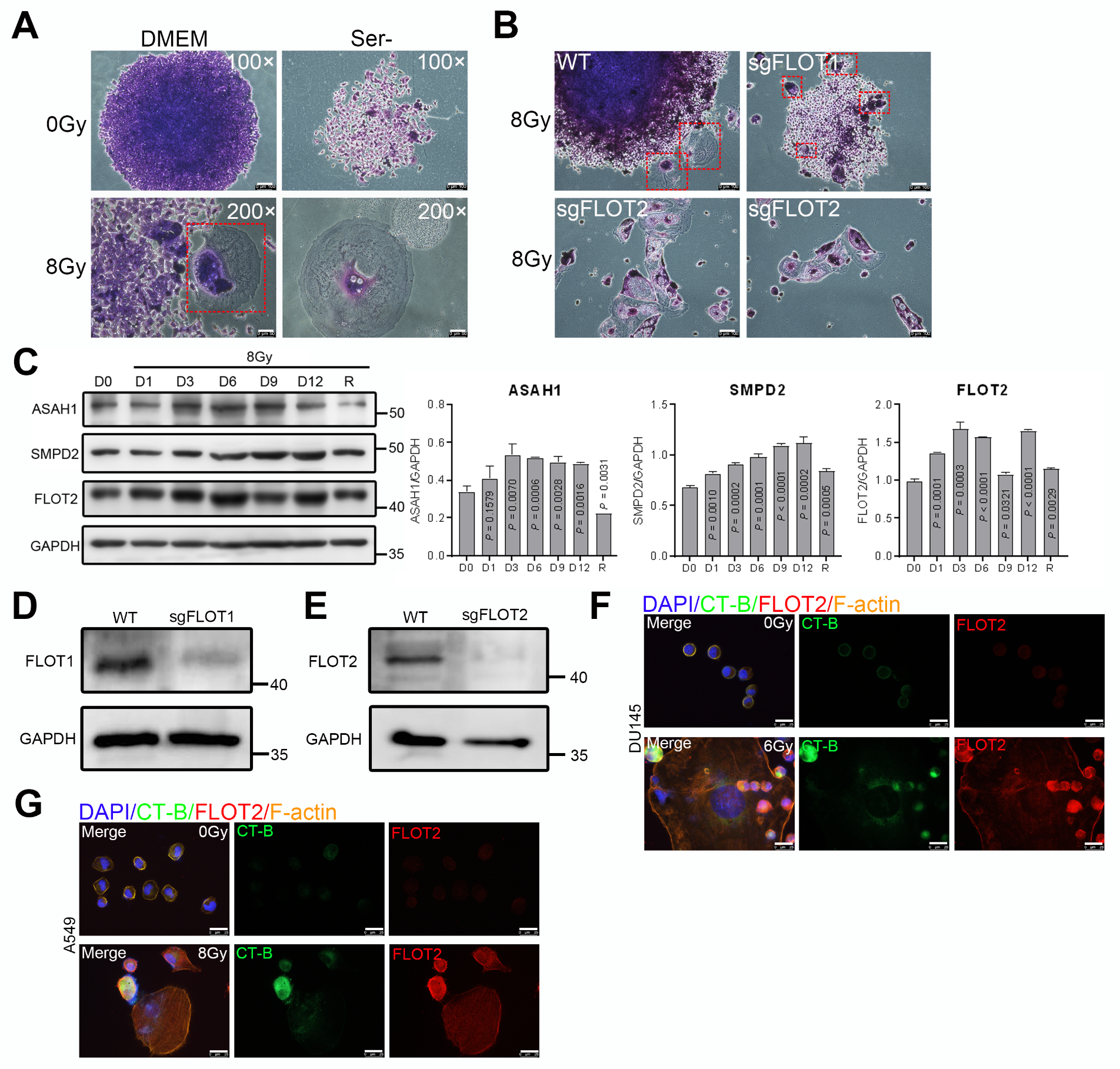


**Figure S3. Flotillin are involved in PGCC budding. A**-**B)** Ineffective budding was observed in HCT116 cells treated with serine deprivation plus 8 Gy (**A**) or FLOT2 knockout plus 8 Gy irradiation (**B**). Radiation-induced PGCCs contributed to colony formation (red dotted box). Scale bars, 100 µm. **C)** Western blot analysis delineates radiation-induced temporal expression profiles of ASAH1, SMPD2, and FLOT2 in HCT116 cells (Left). Corresponding densitometric quantitation is presented as bar graphs (Right). The Day0 group was used as a control for comparison. Student’s t-test. **D**-**E)** Western blot showing remarkably decreased expression or knockout of FLOT1 (**D**) and FLOT2 (**E**) in HCT116 cells. **F**-**G)** Expression of CT-B and FLOT2 in A549 (**F**) or DU145 (**G**) cells with or without radiation treatment. Scale bar, 25 µm.


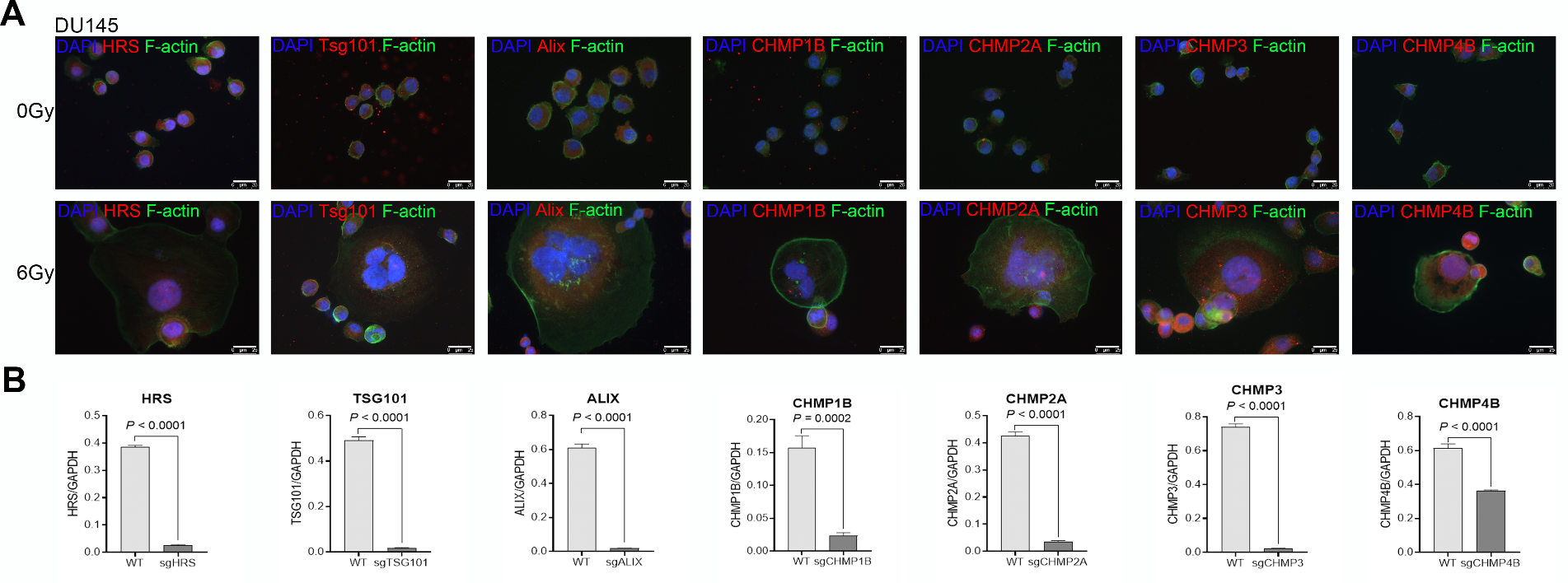


**Figure S4. ESCRT components are involved in PGCC budding. A)** Immunofluorescence staining of HRS, TSG101, ALIX, CHMP2A, CHMP1B, CHMP3, and CHMP4B in the untreated PGCCs and radiation-induced budding PGCCs in DU145 cells. Scale bar, 25 µm. **B)** Bar graphs represent densitometric quantification of Western blot bands obtained from Figure 4C. Statistical significance was assessed using Student’s t-test.


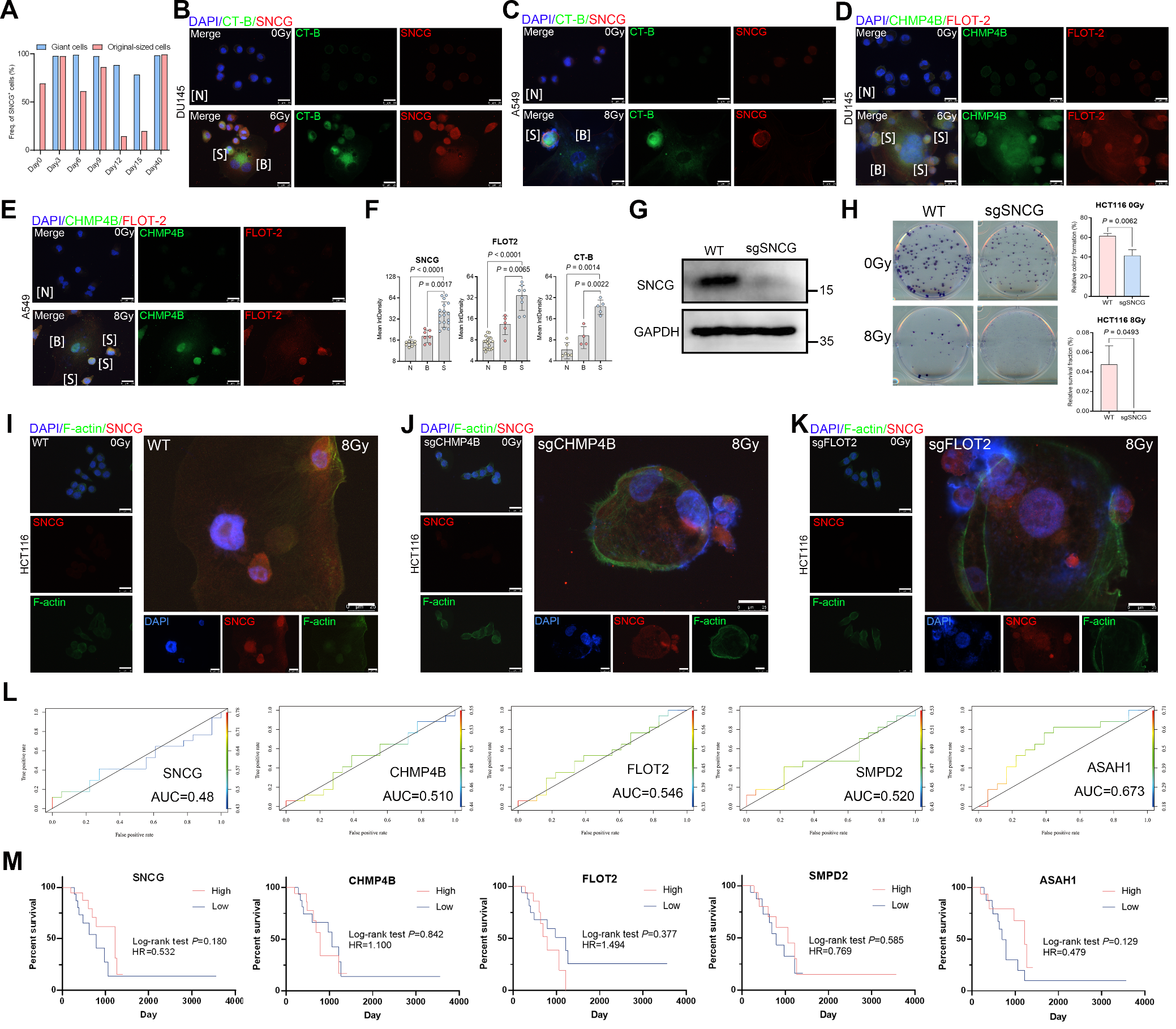


**Figure S5.** **SNCG, FLOT2, and CHMP4B are highly enriched in budded progeny cells. A)** Flow cytometry quantification of SNCG positive rate in both PGCCs and original-sized HCT116 cells. **B**-**C)** Immunofluorescence staining showing the expression of CT-B and SNCG in untreated or budding DU145 (**B**) and A549 (**C**) cells. Scale bar, 25 µm. **D**-**E)** Immunofluorescence staining showing the expression of CHMP4B and FLOT2 in untreated or budding DU145 (**D**) and A549 (**E**) cells. Scale bar, 25 µm. **F)** Bar graphs showing the mean integrated density quantification of immunofluorescence staining from Figure 5D and Figure 5E. Student’s t-test. **G)** Western blot analysis showing minimal SNCG expression in SNCG-knockout HCT116 cells. **H)** Colony formation capacity of wild-type HCT116 and SNCG-knockout cells with or without irradiation (200 cells/well for 0 Gy and 50,000 cells/well for 8 Gy; triplicates for each condition). Mean ± SD; Student’s t-test. **I-K)** Immunofluorescence staining of SNCG in untreated or 8 Gy-induced budding PGCCs and budded progeny cells derived from wild-type HCT116 (**I**), HCT116-sgCHMP4B (**J**), and HCT116-sgFLOT2 (**K**). Scale bar, 25 µm. **L)** ROC curves of SNCG, CHMP4B, FLOT2, SMDP2, and ASAH1 in predicting clinical outcomes of patients with colon and rectal adenocarcinoma (CO/READ) after radiotherapy. **M)** Kaplan–Meier survival curves for progression-free survival (PFS) based on high versus low expression of SNCG, CHMP4B, FLOT2, SMDP2, and ASAH1, plotted according to follow-up time.


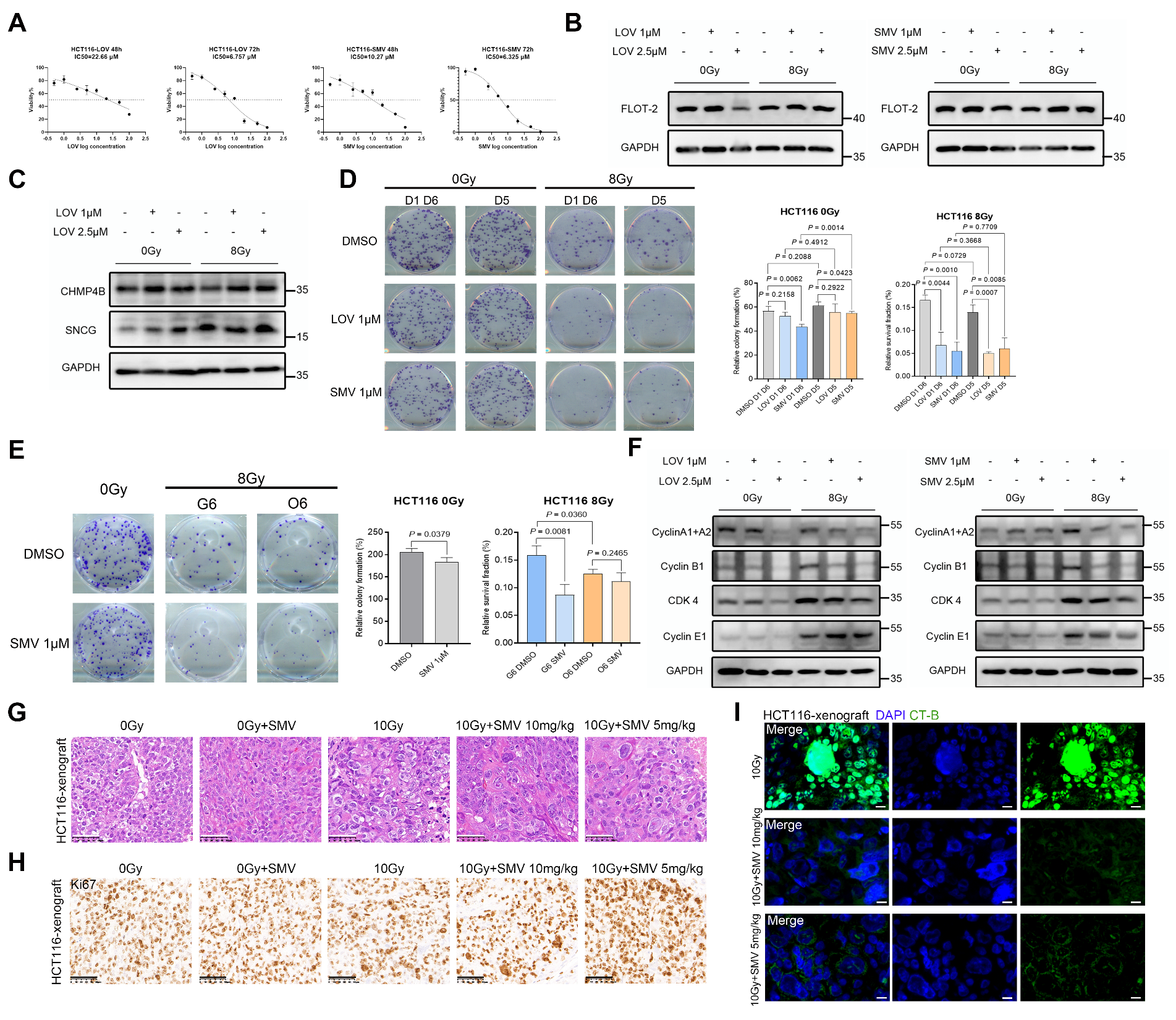


**Figure S6. Low-dose statins did not interfere with cell cycle or flotillin-2 expression but destabilized lipid rafts. A)** Cytotoxicity of lovastatin (LOV) and simvastatin (SMV) in HCT116 cells. The inhibitory concentration (IC50) values on HCT116 cells were tested after 48h or 72h of incubation. **B**-**C)** Western blot assessed the expression of FLOT2, SNCG, and CHMP4B in HCT116 cells after treatment with low-dose statins. **D)** Effects of different doses of statins on colony formation assay (400 cells per well for 0 Gy and 50,000 cells per well for 8 Gy, triplicates for each condition). Statins were added once at day 5 post-irradiation (single-dose group) or twice at days 1 and 6 post-irradiation (two-dose group). Data are presented as mean ± SD, and statistical significance was assessed using Student’s t-test. **E)** Effects of simvastatin (SMV) on colony formation in unirradiated, 8 Gy-induced giant (G6), and original-sized (O6) HCT116 cells. G6 and O6 were sorted on day 6 post-irradiation (400 cells per well for 0 Gy and 50,000 cells per well for G6 and O6, in triplicate). The mean number of colonies ± SD (left panel) and the mean survival fraction ± SD (right panel) are shown. Student’s t-test. **F)** Western blot shows the expression of cell cycle-related protein in HCT116 cells after treatment with low-dose statins. **G**-**H)** H&E (**G**) and Ki67 staining (**H**) show the morphologic changes in 10 Gy-treated and 10 Gy plus statin-treated HCT116 xenografts. Scale bar, 50 µm. **I)** PGCC budding was still observed in 10 Gy-irradiated HCT116 xenograft tumors, but not in 10 Gy plus statin-treated HCT116 xenograft tumors. Scale bar,10 µm.
